# Liraglutide efficacy and action in non-alcoholic steatohepatitis (LEAN): study protocol for a phase II multicentre, double-blinded, randomised, controlled trial

**DOI:** 10.1136/bmjopen-2013-003995

**Published:** 2013-11-04

**Authors:** Matthew J Armstrong, Darren Barton, Piers Gaunt, Diana Hull, Kathy Guo, Deborah Stocken, Stephen C L Gough, Jeremy W Tomlinson, Rachel M Brown, Stefan G Hübscher, Philip N Newsome, on behalf of the LEAN trial team

**Affiliations:** 1 NIHR Liver BRU and Centre for Liver Research, University of Birmingham, Birmingham, UK; 2 Liver and Hepatobiliary Unit, Queen Elizabeth Hospital Birmingham, Birmingham, UK; 3 NIHR Liver BRU Clinical trials group (EDD), CRUK clinical trials unit, University of Birmingham, Birmingham, UK; 4 Newcastle Clinical Trial Unit, Institute of Health and Society, Baddiley-Clark Building, Newcastle University, Newcastle upon Tyne, UK; 5 Oxford Centre for Diabetes, Endocrinology and Metabolism, University of Oxford, Churchill Hospital, Oxford, UK; 6 Centre for Diabetes, Endocrinology and Metabolism, University of Birmingham, Birmingham, UK; 7 Department of Cellular Pathology, Queen Elizabeth Hospital Birmingham, Birmingham, UK; 8 School of Cancer Sciences, University of Birmingham, Birmingham, UK

**Keywords:** Clinical Pharmacology, Histopathology

## Abstract

**Introduction:**

Non-alcoholic steatohepatitis (NASH) is now the commonest cause of chronic liver disease. Despite this, there are no universally accepted pharmacological therapies for NASH. Liraglutide (Victoza), a human glucagon-like peptide-1 (GLP-1) analogue, has been shown to improve weight loss, glycaemic control and liver enzymes in type 2 diabetes. There is currently a lack of prospective-controlled studies investigating the efficacy of GLP-1 analogues in patients with NASH.

**Methods and analysis:**

Liraglutide efficacy and action in NASH (LEAN) is a phase II, multicentre, double-blinded, placebo-controlled, randomised clinical trial designed to investigate whether a 48-week treatment with 1.8 mg liraglutide will result in improvements in liver histology in patients with NASH. Adult, overweight (body mass index ≥25 kg/m^2^) patients with biopsy-confirmed NASH were assessed for eligibility at five recruitment centres in the UK. Patients who satisfied the eligibility criteria were randomly assigned (1:1) to receive once-daily subcutaneous injections of either 1.8 mg liraglutide or liraglutide-placebo (control). Using A'Hern's single stage phase II methodology (significance level 0.05; power 0.90) and accounting for an estimated 20% withdrawal rate, a minimum of 25 patients were randomised to each treatment group. The primary outcome measure will be centrally assessed using an intention-to-treat analysis of the proportion of evaluable patients achieving an improvement in liver histology between liver biopsies at baseline and after 48 weeks of treatment. Histological improvement will be defined as a combination of the disappearance of active NASH and no worsening in fibrosis.

**Ethics and dissemination:**

The protocol was approved by the National Research Ethics Service (East Midlands—Northampton committee; 10/H0402/32) and the Medicines and Healthcare products Regulatory Agency. Recruitment into the LEAN started in August 2010 and ended in May 2013, with 52 patients randomised. The treatment follow-up of LEAN participants is currently ongoing and is due to finish in July 2014. The findings of this trial will be disseminated through peer-reviewed publications and international presentations.

**Trial registration:**

clinicaltrials.gov NCT01237119.

## Introduction

Non-alcoholic fatty liver disease (NAFLD) is now the commonest cause of chronic liver disease, affecting up to 30% of the general population^1–3^ and 70–90% of high-risk individuals.^3^ ^4^ This prevalence relates to the dramatic rise in recent years of morbid obesity and type 2 diabetes (T2D). Even though simple hepatic steatosis (without fibrosis) is arguably a benign condition, up to a quarter of patients with NAFLD have the more severe, inflammatory condition known as non-alcoholic steatohepatitis (NASH).^5^ Patients with NASH have an increased risk of progression to cirrhosis, liver failure and hepatocellular carcinoma,^6^ and are expected to become the commonest indication for liver transplantation in forthcoming years.^7^ Despite this, there are no universally accepted pharmacological therapies for NASH. Therefore the need for novel, safe agents in NASH is of paramount importance to prevent disease progression and the accompanying clinical burden.

The strong association of NASH with metabolic syndromes, in particular central adiposity and insulin resistance, provides a strong rationale for investigating therapies that induce weight loss and insulin sensitivity. The gut-derived incretin hormone, glucagon-like peptide-1 (GLP-1), is therefore an attractive target option in NASH. Native GLP-1 has a potent blood glucose-lowering action mediated through its ability to induce insulin secretion and reduce glucagon secretion in a glucose-dependent manner, as well as suppressing appetite and slowing gastric emptying.^8^ Human GLP-1, however, only has a short half-life (1.5–2 min) as it is rapidly degraded by the enzyme dipeptidyl peptidase-4 (DPP-4).^9^ Liraglutide (Victoza) is a long-acting (half-life 13 h) GLP-1 analogue with 97% structural homology to the native hormone and is administered once daily by subcutaneous injection.^10^ Liraglutide has been shown to cause dose-dependent weight loss,^11^ ^12^ decrease glycosylated haemoglobin (HbA1c), systolic blood pressure and improve β-cell function.^13–18^ Subsequently, it has been licensed for glycaemic control in overweight patients with type 2 diabetes (T2D).^19^ There is, however, a paucity of data in patients with liver disease, and in particular histologically defined NASH.

GLP-1 analogues, including liraglutide, have been shown to improve liver enzymes, oxidative stress and hepatic steatosis in murine models in vivo and in isolated in vitro murine and human hepatocyte studies.^20–25^ To date, human studies investigating the effect on liver injury have been limited to case reports,^26^ ^27^ solitary case series (n=8)^28^ and retrospective (*liver enzyme*) studies in patients with T2D.^29^ A large meta-analysis of six phase III randomised controlled trials (RCT), that comprised the Liraglutide Effect and Action in Diabetes (LEAD) programme (>4000 patients), highlighted that the 26-week treatment with 1.8 mg once daily liraglutide was well-tolerated and resulted in significant improvements in liver enzymes compared to placebo control in overweight patients with T2D.^30^ However, limitations of this study were the retrospective nature of its analysis and the lack of any liver biopsy data.

On this basis, we hypothesised that the 48-week treatment with liraglutide would result in significant improvements in liver histology in overweight patients with NASH. To test this hypothesis, we designed a phase II, multicentre, double-blinded, placebo-controlled RCT, entitled ‘Liraglutide Efficacy and Action in NASH (LEAN).’

## Methods

### Study design overview

LEAN is a 48-week multicentre, double-blinded, placebo-controlled randomised clinical trial of treatment with the once daily human GLP-1 analogue, liraglutide (Victoza), for adults with biopsy-proven NASH. Screening was undertaken within 14 days of randomisation to assess eligibility and collect baseline data. Patients who satisfied the eligibility criteria were randomly assigned (1:1) to receive subcutaneous injections once daily of either 1.8 mg liraglutide (experimental) or liraglutide-placebo (control). After which, a 12-week washout period is scheduled.

**Figure 1:**
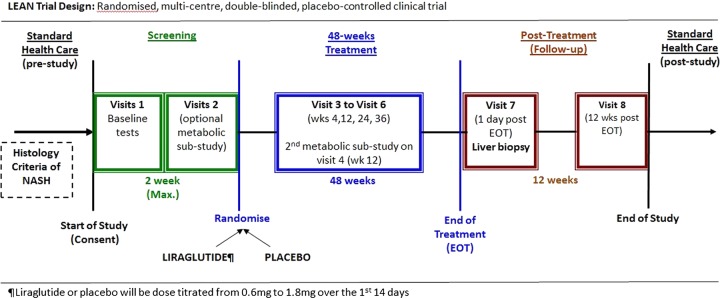
Schematic of liraglutide efficacy and action in non-alcoholic steatohepatitis trial design. Eligible participants are randomly assigned to a 48-week treatment of once daily subcutaneous injections of either 1.8 mg liraglutide or placebo control. Both the trial investigators and the participants are blinded to drug allocation.

The primary outcome measure will be assessed using an intention-to-treat analysis of the proportion of evaluable patients achieving an improvement in liver histology between liver biopsies at baseline (within 6 months of screening) and after 48 weeks of treatment. Histological improvement will be defined as a combination of the disappearance of active steatohepatitis (ie, disappearance of hepatocyte ballooning) and no worsening in fibrosis (Kleiner Fibrosis score^31^). A schematic of the trial design is summarised in figure 1.

### Ethical and regulatory approval

The National Research Ethics Service (NRES) East Midlands—Northampton committee (previously known as Leicestershire, Northamptonshire and Rutland Research Ethics Committee, UK) and the Medicines and Healthcare products Regulatory Agency (MHRA) approved all versions (including current V.7.0) of the study protocol. In addition, all five recruitment sites obtained approval from their respective hospital Research and Development (R&D) departments prior to the start of screening.

### Treatment groups

Patients who satisfied the eligibility criteria were randomly assigned on a 1:1 basis to a 48-week treatment of either liraglutide (Victoza; 1.8 mg once daily) or liraglutide-placebo control (1.8 mg once daily).

#### Liraglutide (active experimental group)

Liraglutide (Victoza, Novo Nordisk A/S, Bagsvaerd, Denmark) was supplied in a cartridge contained in a prefilled multidose disposable pen. Each prefilled pen contained 18 mg liraglutide in 3 mL of clear, colourless, isotonic solution (including water for injections, disodium phosphate dehydrate, propylene glycol and phenol). Liraglutide was administered once daily, at any time of the day, as a single subcutaneous injection into the abdomen, thigh or upper arm using the prefilled pen (30 or 31 gauge needles). Participants were encouraged to inject liraglutide at the same time each day, according to the most convenient time for them. Participants were instructed to perform an air shot of 0.2 µL before the first use of each new prefilled pen to ensure that it functioned correctly.

To improve gastrointestinal tolerability participants underwent a 14-day dose-titration period in keeping with previous reports.^13–18^ The dose was titrated by 0.6 mg every 7 days from a starting dose of 0.6 mg once daily until the maximum dose of 1.8 mg once daily was achieved. Prior to the current trial design, no study had investigated any form of GLP-1-based therapy in patients with biopsy-confirmed NASH or any other form of liver disease. Therefore, the rationale for using a dose of 1.8 mg once daily was based on previous reports in overweight patients with or without T2D.^13–18^ Furthermore, a large meta-analysis of six phase III clinical trials (LEAD programme) of liraglutide therapy for poorly controlled T2D found that patients with abnormal liver transaminases had a similar drug safety profile to those with normal liver transaminases. In addition, greater improvements in liver transaminases and CT-measured hepatic steatosis were seen with 1.8 mg liraglutide than 1.2 and 0.6 mg doses.^30^

#### Liraglutide-placebo (inactive, placebo-control group)

Liraglutide-placebo (Victoza, Novo Nordisk A/S, Bagsvaerd, Denmark) was packaged, administered and dose-titrated in an identical manner to the liraglutide comparator, described above. The composition of the placebo solution for injection was identical to its comparator, with the exclusion of the active liraglutide substance. A placebo was used to provide an assessment of the level of response with an injectable placebo, which could potentially be higher than that seen with oral placebo agents.

#### Concomitant therapy

No dose reductions of liraglutide or placebo were allowed throughout the 48-week treatment period. Previous treatment with oral antidiabetic drugs (metformin and/or sulfonylurea) was continued at the same dose in participants with T2D at randomisation. In the event of recurrent major hypoglycaemic episodes (requiring medical or hospital intervention), the dose of the sulfonylurea was reduced by 50% at the discretion of the investigators. The reported rate of hypoglycaemia in literature, with liraglutide monotherapy or in combination with metformin, is very low.^13–18^ However in the event of recurrent major hypoglycaemic episodes in which no dose reduction could be undertaken (ie, not on a sulfonylurea) the participant was withdrawn from treatment at the discretion of the chief investigator.

Glycaemic control was assessed at each 12-weekly trial visit with self-measured plasma glucose readings and HbA1c. In the event that glycaemic control deteriorated, defined as HbA1c >9% (75 mmol/mol), the participant was informed and counselled with regard to starting open-labelled long-acting insulin detemir once daily (Levemir). However, the patient's participation in the trial was not jeopardised if they did not wish to start insulin detemir. The insulin detemir dose was titrated by trial investigators in accordance with European guidelines (http://www.ema.europa.eu) to ensure that the participant's standard of diabetes care was not significantly compromised as a result of participating in the clinical trial. The HbA1c cut-off >9% was based on the opinions of the TMG (MJA and PNN), consisting of expert endocrinologists (SCLG and JWT), and in accordance with previous clinical trial guidance.^32^

In addition to study medications, participants continued to receive standard National Health Services (NHS) care recommendations concerning life-style modifications (ie, exercise, weight loss and dietary modification) and management of various coexisting illnesses throughout the trial. Patients were asked to limit alcohol consumption to less than 20 mg/day for women (ie, 14 units/week) and 30 mg/day for men (ie, 21 units/week). These levels were consistent with the UK Department of Health recommended daily alcohol allowance (British Medical Association 1995). Participants were not allowed any new prescription or over-the-counter therapies (ie, herbal remedies, milk thistle) that may improve or worsen NASH throughout the duration of the trial. Potential NASH therapies that were not allowed during the trial duration included thiazolidinediones (TZDs), DPP-4 inhibitors, other GLP-1 receptor agonists (eg, exenatide), vitamin E and orlistat. Steroids (oral or intravenous), methotrexate and/or amiodarone were also not permitted based on their ability to promote hepatic steatosis.

### Outcome measures

#### Primary outcome measure

The primary outcome measure is the proportion of participants with a significant improvement in liver histology between liver biopsies at baseline (ie, within 6 months of screening) and at the end of a 48-week treatment. The definition of a significant histological improvement requires both the disappearance of steatohepatitis (defined as a disappearance of hepatocyte ballooning) and no worsening of fibrosis, as assessed by the Kleiner scoring system.^31^ Hepatocyte ballooning is now widely recognised as the key lesion for distinguishing NASH from simple steatosis.

#### Secondary outcome measures

Secondary outcome measures include changes in; (1) overall NAFLD Activity Score (NAS)^31^; (2) individual histological components of NAS, including lobular inflammation, steatosis, hepatocyte ballooning and fibrosis; (3) serum markers of steatosis (SteatoTest), NASH (NashTest, caspase-cleaved cytokeratin-18 (CK-18 M30)) and fibrosis (Enhanced Liver Fibrosis (ELF; iQUR Ltd), FibroTest); (4) liver stiffness evaluation (LSE) with Transient Elastography (Fibroscan, Echosens, Paris, France); (5) insulin resistance (HOMA-IR); (6) anthropometric measures including body weight, body mass index (BMI) and waist circumference; (7) lipid profile and glycaemic control (HbA1c, fasting plasma glucose); (8) serum alanine aminotransferase levels and (9) health-related quality of life (QOL; SF-26 V.2.0) and nutrition (Block Brief 2000 Food Frequency Questionnaire (FFQ) questionnaires).

### Analytical methods

#### Liver histopathology

**Figure 2:**
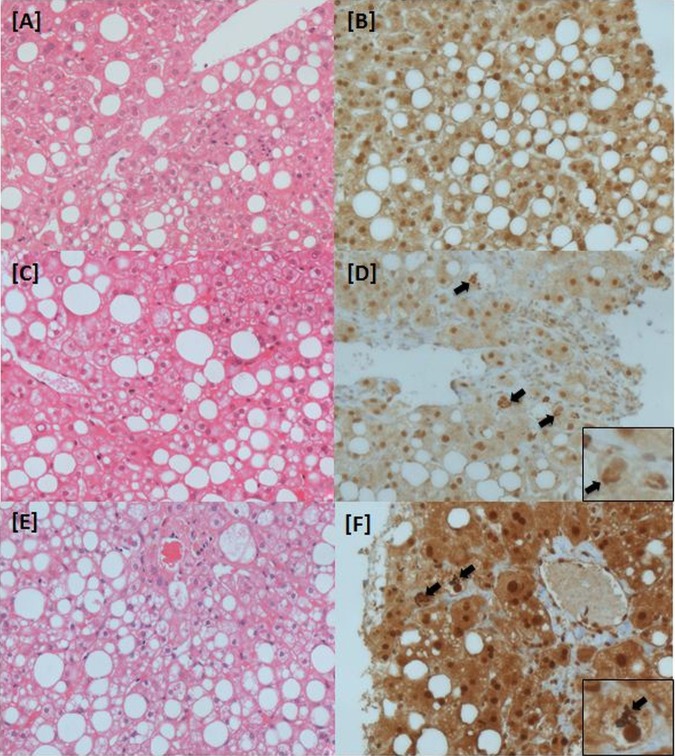
Histological inclusion criteria for liraglutide efficacy and action in non-alcoholic steatohepatitis (LEAN) trial. Liver biopsy sections (actual magnification ×400). (A and B) ‘Uncertain’ non-alcoholic steatohepatitis (NASH)—not eligible for LEAN: (A) H&E stain highlights fat, inflammation and some pale cells, however (B) ubiquitin immunohistochemistry does not identify any Mallory Denk bodies (no confirmed ballooning). (C and D) ‘Uncertain’ NASH—eligible for LEAN: (C) H&E stain highlights fat, inflammation and pale cells, but with no obvious Mallory Denk bodies. However, ubiquitin staining (D) is positive (confirming ballooned hepatocytes). (E,F) ‘Definite’ NASH—eligible for LEAN: both H&E and ubiquitin staining highlight fat, lobular inflammation and widespread ballooned hepatocytes. Black arrows highlight Mallory Denk bodies.

Two independent liver histopathologists (SGH and RMB) at the central trial site (Birmingham, UK) will perform all the histopathological assessments using an in-house designed proforma (see online supplementary table S1). Both histopathologists will be blinded to the clinical, laboratory and study treatment allocation. The histological diagnosis of NASH will be established using H&E staining and haematoxylin van Gieson stains of formalin-fixed paraffin-embedded liver tissue. Both the baseline and end-of-treatment (48 weeks) biopsies will be reported as either ‘definite NASH,’ ‘uncertain NASH’ or ‘not NASH.’ The histological diagnosis of ‘definite NASH’ is defined as a combination of >5% macrovesicular steatosis, hepatocyte ballooning (± Mallory's Hyaline) and lobular inflammation (mixed infiltrate, related to foci of ballooning).^33^ The assessment of ballooning is subjective, and thus for ‘uncertain’ hepatocyte ballooning, a key component of the diagnosis of NASH, ubiquitin immunohistochemistry will be used to identify material compatible with Mallory's hyaline (figure 2). To validate the quality of the biopsy specimen, the core specimen length will be measured and the number of portal tracts will be recorded.

The NAS will be calculated based on the Kleiner classification.^31^ The NAS is scored of 8, with 8 representing the highest activity. The NAS is the sum of scores of the 3 components of the histological scoring system, namely steatosis (0=<5%, 1=5–33%, 2=>33–66%, 3=>66%), lobular inflammation (0=no foci, 1=<2 foci/×200, 2=2–4 foci/×200, 3=>4 foci) and hepatocyte ballooning (0=none, 1=few ballooned cells, 2=many cells/prominent ballooning). The Kleiner scoring system for NAFLD fibrosis (F0-F4)^31^ and a modified version of the Ishak score^34^ (F0-F6; see online supplementary table S1) will be used to evaluate the stage of fibrosis in each biopsy specimen. The Ishak score was modified from the original scoring system, reported in 1995,^34^ in order to include the zone 3 peri-cellular/peri-sinusoidal fibrosis, which is characteristically seen in NASH. Portal tract changes (inflammation, interface hepatitis, ductular reaction), an intrinsic feature of NASH, will also be recorded.^35^

The pathologists will assess the biopsies independently and fill in separate forms. Cases where there is disagreement on the classification, as ‘NASH’ or ‘not NASH,’ will be reviewed and a consensus opinion given. Also discrepancies of more than 1 point on any of the scoring scales (NAS, Kleiner fibrosis scoring system and modified Ishak score) will be reviewed and an amended consensus view offered. Discrepancies of only 1 point will not be altered.

#### Clinical and laboratory data

Fasting blood samples will be analysed for full blood count, urea, creatinine and electrolytes, thyroid stimulating hormone (TSH), lipid profile (total cholesterol, high-density lipoprotein, triglycerides), liver function tests, prothrombin time, international normalised ratio (INR), amylase, α-fetoprotein, C reactive protein, HbA1c, calcitonin and plasma glucose using standard laboratory methods (Roche Modular system, Roche Ltd, Lewes, UK). Serum Insulin (Mercodia, Uppsala, Sweden), non-esterified fatty acids (Zen-Bio, Research Triangle Park, North Carolina, USA) and CK-18 M30 (M30 Apoptosense ELISA Kit; PEVIVA AB, Bromma, Sweden) will be measured in-house using commercially available colorimetric ELISAs. Serum caspase-cleaved cytokeratin-18 (CK-18 M30) and the ELF Test were performed at study entry to assess hepatic apoptosis and fibrosis, respectively. The FibroMax panel (consisting of the SteatoTest, NashTest, FibroTest) will be undertaken by Lab 21 Ltd (Cambridge, UK). The ELF test, which combines three direct serum markers of fibrosis (hyaluronic acid, pro-collagen III amino terminal peptide and tissue inhibitor of metalloproteinase 1) using an algorithm developed by the European Liver Fibrosis Group,^36^ will be performed on fasting serum stored at −80° by a commercial laboratory (iQUR Ltd, Royal Free Hospital, London, UK).

T2D was considered present if patients had a recorded diagnosis in their medical records or if the fasting plasma glucose was ≥7 mmol/L and/or if the 2 h 75 g oral glucose tolerance test (OGTT) plasma glucose was ≥11.1 mmol/L. All patients without a recorded history of T2D were screened with an OGTT. Impaired glucose tolerance (IGT) was defined as a 2 h plasma glucose between 7.8 and 11.1 mmol/L. HOMA-IR, a marker of insulin resistance, was calculated in the standard fashion: glucose×insulin/22.5.

Measurements of weight (kg), height, systolic/diastolic blood pressure and waist:hip circumferences were recorded. Waist and hip circumferences were defined as the circumferential measurements immediately above the level of the iliac crests and at the level of the greater trochanters, respectively. BMI was defined as weight in kilograms divided by the square of the height in metres (kg/m^2^).

LSE was measured using Transient Elastography (Fibroscan, Echosens, France). The median value and IQR of 10 validated measurements were recorded within the range of 2.5–75 kPa. The XL probe was used on individuals who have a BMI greater than 30 kg/m^2^ or when the Fibroscan 502 Touch machine (automated) recommends its use over the M-probe. To achieve a valid LSE (median of successful liver stiffness measurements) the operator had to obtain all of the following three criteria: (1) ≥10 successful liver stiffness measurements; (2) IQR/median ratio <0.30 and (3) ≥60% measurement success rate.^37^

#### Patient questionnaires

QOL was assessed by the Short Form 36 V.2.0 (SF-36v2) health-related QOL questionnaire (QualityMetric Health Outcomes Solutions, Lincoln, USA). The SF-36v2 questionnaire was a practical, reliable and valid measure of physical and mental health that could be completed in 5–10 min. It consisted of 36 questions that assessed the functional health and well-being from the study participant's point of view.^38^ The Block Brief 2000 FFQ (Block Dietary Data Systems, California, USA) was completed by each participant to assess the usual and customary intake of a wide array of nutrients and food groups. The food list incorporated in the Block questionnaire was developed from the National Health and Nutrition Examination Survey III dietary recall data. The Block Brief 2000 FFQ consisted of a well-validated self-administered questionnaire consisting of 70 food-related questions and took approximately 15 min to complete.^39^ Pictures of standardised serving sizes and an American-to-English food translation sheet (ie, ‘Catsup’=tomato ‘Ketchup’) were used to aid completion of the questionnaire.

The Alcohol Use Disorder Identification Test (AUDIT) questionnaire was used to assess the frequency of alcohol consumption and screen out alcohol-related problems, and dependence symptoms.^40^ The AUDIT questionnaire consisted of a 10-item questionnaire that took 2–5 min to complete. The questionnaire has a positive predictive value of 98% for hazardous drinking, and a negative predictive value of 97% for alcohol dependence. The overall score ranges from 0 to 40, with a score of less than 8 being a good indication of insignificant alcohol consumption.

All questionnaires were completed at baseline (visit 1), end-of-treatment (visit 7) and 12 weeks post-treatment (visit 8). Analysis will report the change from baseline scores to both the end-of-treatment and follow-up scores.

### Statistical justification and outcome analysis

#### Sample size justification

This is an early phase II trial randomising patients equally between two treatment arms—one experimental (liraglutide) and one control (placebo). The primary aim is not to determine the efficacy of liraglutide compared with placebo but to assess whether the efficacy and safety profile of liraglutide is worthy of further investigation. Recruiting patients into a no-treatment control group provides simultaneous unbiased assessment of comparable patient groups.

At the time of the study design there were no available data to estimate histological response with a 48-week treatment of liraglutide (Victoza). Based on previous non-GLP-1 pharmaceutical trials in NASH utilising improvements in liver histology as a primary endpoint,^41^ ^42^ it was assumed that 14–17% of patients undergoing current standard of care (placebo) would have an improvement in NASH by week 48. It was estimated that 20% of the placebo-control arm would achieve an improvement in liver histology, based in part on the knowledge that the placebo effect might be exaggerated by the subcutaneous injection route of administration (vs oral route in previous NASH trials) in the current trial. To justify further investigation of liraglutide treatment, a clinically relevant improvement in liver histology was required in at least 50% of patients. The sample size was calculated using A'Hern's single stage phase II methodology,^43^ with a significance level of 0.05 (type 1 error) and power of 0.90 (type II error). The design required 21 evaluable patients in the treatment group. The published literature in NASH trials reported on average a participant withdrawal rate of 10–20%.^41^ ^42^ ^44^ Therefore, to account for a 20% withdrawal rate the recruitment target was inflated from 21 to 25 patients per treatment group; the total recruitment target being 50 patients randomised in a 1:1 allocation ratio to either liraglutide or placebo.

### Analysis of outcome measures

All evaluable patients will be analysed on the intention-to-treat principle. Evaluable patients are defined as those who have had an end-of-treatment biopsy (visit 7), irrespective of the amount of treatment they have received. Patients will be categorised as either achieving the primary histological endpoint (ie, disappearance in NASH) or not. The proportion of patients with a reported improvement in liver histology will be presented and compared across treatments descriptively with 95% CIs. The proposed A'Hern's design stipulates that 8 or more evaluable patients of 21 in the experimental treatment group (liraglutide) need to achieve the defined improvement in liver histology for treatment with liraglutide to be deemed worthy of further investigation with a phase III trial. Analyses will be presented for the subgroups of patients with and without T2D. Patients who have not had an end-of-treatment biopsy will be classed as non-evaluable and will not be included in the primary analysis.

Secondary analysis of the primary outcome measure will report (1) the number and proportion of patients that did not have an end-of-treatment biopsy and the reasons for this (these will be classified as ‘no histological improvement’) and (2) the number and proportion of patients that were considered to have had sufficient treatment and an end-of-treatment biopsy. In addition, an analysis that directly compares the two proportions for the separate treatment arms will be performed using the χ^2^ test.

Secondary measures collected as continuous and categorical variables will be presented with 95% CIs descriptively across treatments using medians and proportions, respectively. Secondary measures collected as longitudinal data (including QOL data, scored as per the questionnaire-specific scoring manuals) will be presented descriptively across treatment groups as changes over time. A summary of all adverse events (AEs) experienced by patients in both arms will be reported.

## Conduct of the trial

### Patient selection

Eligible adults (≥18 years old) were identified and recruited at the participating trial site centres starting in August 2010 and by May 2013, 52 patients were recruited. Participating UK trial centres included the liver units at the Queen Elizabeth University Hospital (Birmingham, from August 2010), Queens Medical Centre (Nottingham, from May 2011), Southampton General Hospital (Southampton, September 2011), Hull Royal Infirmary (Hull, November 2011) and St James Hospital (Leeds, from May 2012). All trial participants gave informed written consent at the beginning of the screening visit prior to undergoing any tests and procedures needed to assess eligibility.

Eligibility for the trial was determined at screening visit 1 by standard blood tests, clinical history (including written-confirmation of drug history where necessary) and physical examination/observations to identify other illnesses or contraindications for participation (trial schedule figure). In addition, after receiving formal training the patient's ability to understand and self-administer the subcutaneous injections using the prefilled treatment pen was assessed by an experienced nurse specialist at screening visit 2. Patients who satisfied the eligibility criteria for the 48-week treatment trial at the Birmingham site were given the option to participate in a metabolic mechanistic substudy. The substudy involved two overnight admissions (approximately 22 h) to the Wellcome Trust Clinical Research Facility (Birmingham) to undergo a two-step hyperinsulinaemic euglycaemic clamp with stable isotopes and adipose microdialysis on visits 2 (pretreatment) and 4 (12-week treatment). A detailed summary of the metabolic substudy will be published separately. A patient's decision to partake or withdraw from the metabolic substudy did not affect their participation in the main 48-week trial.

#### Inclusion criteria

The trial entry criteria were based on a diagnosis of ‘definite’ NASH on liver biopsy obtained within 6 months of screening. Prior to randomisation, two independent liver histopathologists (SGH and RMB) from the central trial site (University Hospital Birmingham, UK) reviewed all of the liver biopsies (internal and external trial sites) of the potential participants to assess whether a diagnosis of ‘definite’ NASH was present. A ‘definite’ diagnosis of NASH was defined if all of the following were present on biopsy: (1) macrovesicular steatosis (>5%); (2) hepatocyte ballooning (± Mallory Hyaline) and (3) lobular inflammation (mixed infiltrate, related to foci of ballooning). The two independent histopathology case report forms (CRFs) were reviewed by a trial investigator (MJA) and in the event that the histopathologists disagreed with regard to the diagnosis of NASH (ie, one judged ‘uncertain’ and the other ‘definite’) a combined histopathology assessment was undertaken to determine the patient's eligibility status. Only patients with ‘definite’ NASH (either on two independent reports or after joint review) were classified as eligible.

All participants had to be ≥18 to <70 years of age and have a BMI ≥25 kg/m^2^ at screening. Patients with T2D mellitus at screening had to have stable glycaemic control (HbA1c <9%) and be managed by either diet and/or a stable dose of metformin/sulfonylurea. Patients without a history of T2D prior to the screening visit underwent OGTT at screening to determine their glycaemic status and were labelled as ‘non-diabetic’ if one or more of the following was confirmed

- Impaired fasting glucose, defined using the European Criteria between 6.1 and 6.9 mmol/L.
- IGT, defined as 2 h plasma glucose levels between 7.8 and 11.0 mmol/L on the 75 g OGTT.
- Normal Fasting Plasma Glucose <6.1 mmol/L and normal 2 h plasma glucose levels <7.8 on the 75 g OGTT.

#### Exclusion criteria

A detailed summary of the exclusion criteria is provided in box 1. In brief, patients with a history or current significant alcohol consumption, poor glycaemic control (HbA1c >9.0%), Child's Pugh B or C cirrhosis or other liver disease aetiology were excluded. The latter was confirmed with a full liver aetiology screen (drug induced, viral hepatitis B/C, autoimmune and genetic) at the screening visit. Past and current alcohol consumption was ascertained by a detailed review of the patient's medical, social history and by a self-administered AUDIT questionnaire with reference pictures to remind participants of drink equivalents. Concomitant use of drugs reported to be inducers (methotrexate, amiodarone, steroids) or potential therapies for NASH (TZDs, vitamin E), or other known hepatotoxins were assessed during the screening visit (box 1). In keeping with previous clinical trials assessing GLP-1 therapies, patients with a history of acute/chronic pancreatitis (of any cause), pancreatic and thyroid carcinoma, and/or a family history of medullary thyroid carcinoma were also excluded.

Box 1 **Exclusion criteria for liraglutide efficacy and action in non-alcoholic steatohepatitis trial**

*Generic exclusion criteria*

1. Refusal or lacks capacity to give informed consent to participate in the trial.
2. Participation in any clinical trial of an investigational therapy or agent within 3 months of randomisation.
3. Patient (or carer) deemed not competent at using the correct site and technique for subcutaneous injection of the trial treatment (containing dummy drug on practice).
4. NAFLD Activity Score (NAS) <3 on liver biopsy.
5. Child's B or C cirrhosis.
6. Medical history of multiple drug allergies (defined as anaphylactoid drug reactions in >2 drug groups).
7. Presence of any acute/chronic infections or illness that at the discretion of the chief investigator might compromise the patient's health and safety in the trial.
8. Pregnancy or breastfeeding.
9. Women, of childbearing age, who are not willing to practise effective contraception (ie, barrier, oral contraceptives, impenon or past medical history of hysterectomy) for the 48-week duration of the trial and for 1 month after the last administration of the drug.
10. Men, sexually active with women of childbearing age, who are not willing to practise effective contraception for the 48-week duration of the trial and for 1 month after the last administration of the drug.
11. Liver disease of other aetiologies (ie, drug-induced, viral hepatitis, autoimmune hepatitis, PBC, PSC, haemochromatosis, A1AT deficiency, Wilsons disease).
12. Medical/surgery history of; gastric bypass surgery, orthotopic liver transplant (OLT) or listed for OLT, hepatocellular, pancreatic, thyroid carcinoma, multiple endocrine neoplasia syndrome type 2 (MEN 2), acute or chronic pancreatitis and total parenteral nutrition within 6 months of randomisation.
13. Diagnosis of malignancy within the last 3 years (with the exception of treated skin malignancies).
14. Hepatocellular carcinoma: dysplastic or intermediate nodules to be excluded. Borderline cases to be discussed at Birmingham's tertiary hepatobiliary multidisciplinary team (MDT) meeting. Regenerative and other nodules to be included at the discretion of the chief investigator and the MDT.
15. Family history of medullary thyroid carcinoma.
16. Clinical evidence of decompensated chronic liver disease: radiological or clinical evidence of ascites, current or previous hepatic encephalopathy and evidence of portal hypertensive haemorrhage or varices on endoscopy.
17. Abnormal clinical examination of thyroid (ie, unexplained goitre or palpable nodules).
18. Alanine aminotransferase or aspartate aminotransferase >10×upper limit of normal.
19. Average alcohol consumption/week male >21 (approximately 210 g), female >14 units (approximately 140 g) within the last 5 years.
20. >5% weight loss since the diagnostic liver biopsy was obtained.
21. Recent (within 3 months of the diagnostic liver biopsy or screening visit) or significant change (as judged by the chief investigator) in dose of the following drugs: inducers of hepatic steatosis (steroids (oral/intravenous), methotrexate, amiodarone), orlistat and/or multivitamins/vitamin E (containing >200% recommended daily amount; >30 mg/day).
22. Known positivity for antibody to HIV.
23. Serum creatinine >150 μmol/L or currently being treated with renal replacement therapy (ie, haemodialysis or peritoneal dialysis).

*Specific exclusion criteria for participants with T2D*

1. Current or previous insulin therapy, with exception of previous short-term insulin treatment in connection with intercurrent illness is allowed (≥3 months prior to screening), at the discretion of the chief investigator.
2. Participants receiving thiazolidinediones (TZDs), dipeptidy peptidase (DPP) IV inhibitors and other GLP-1-based therapies (ie, exenatide).
3. HbA1c ≥9%.
4. Recurrent major hypoglycaemia or hypoglycaemic unawareness as judged by the chief investigator.

Patients who met any of the criteria (*listed* above) at the screening visit were excluded from trial participation.

### Randomisation

Participants who met all the eligibility criteria and provided written informed consent were randomly assigned on a 1:1 basis to either of the two-study treatments (liraglutide vs placebo) using computer generated randomisation at the Cancer Research UK Clinical Trials Unit (CRCTU). The randomisation was stratified to ensure that there were equal numbers of patients with/without T2D in each treatment group and that each trial site had equal numbers of patients on each treatment. Trial participants were allocated a unique trial identification number to preserve patient confidentiality and enable the study to be double-blinded.

### Medication preparation and blinding/unblinding procedures

Both liraglutide and placebo control were packaged and labelled with a unique identification number (in keeping with the European Unions Good Manufacturing Practice for Medicinal Product guidelines) by the manufacturer (Novo Nordisk Ltd), to the extent that the receiving trial site was blinded to the study drug throughout the duration of the trial. Sealed parcels (containing electronic information) were sent with each drug package for the attention of the unblinded members of the central trial management group (TMG) nominated statistician, PG and database programmer, PM, to ensure (1) safe delivery of the correct drug and (2) blinding of the treatment allocation from the remainder of the TMG and the trial patient. An independent unblinding service (24/7) was provided by the Medical toxicology and Information services, Guys hospital (London, UK), throughout the duration of the trial.

Unblinding of treatment only takes place if the identity of the allocated study medication was necessary for patient safety and care. If a serious adverse event (SAE) was deemed unexpected and possibly, probably or definitely related to liraglutide (ie, suspected unexpected serious adverse reaction=SUSAR), a clinical member of the TMG was unblinded to the medication to evaluate causality. Subsequently, the event was either labelled as an unrelated SAE (for patients receiving placebo) or a SUSAR (for patients receiving liraglutide). The latter were reported to the MHRA and the NRES, and only if patient safety was jeopardised was the study medication discontinued and the treating clinician/patient informed.

### AE reporting and analysis

The reporting period for AEs started at screening visit 1 and continued until follow-up visit 8. SAEs were reported until day 336 (week 48) of the trial treatment and for 30 days post-EOT. All SAEs and adverse reactions were evaluated by the investigators and recorded. The National Cancer Institute's common terminology criteria for AEs (CTCAE, V.4.02, 2010) was used to grade each AE. The central trial office (CRCTU, Birmingham) kept detailed records of all AEs reported (nature, onset, duration, severity, outcome) and performed an evaluation with respect to seriousness, causality and expectedness. Interim analysis of safety data was performed and presented to the independent data management committee (DMC) on a 6-monthly basis. The unblinded DMC advised accordingly with regards to the trial conduct and specifically whether extra/new data monitoring was required for the remainder of the trial. The DMC operated in accordance with a trial specific charter based on the template created by the Damocles Group. Specific attention was given to AEs related to the thyroid (measures of blood calcitonin, TSH and physical examination) and pancreas (blood amylase, symptom recognition for pancreatitis), in light of previous non-human (rodents) and postmarketing human safety data (in patients with diabetes), respectively.^45^ ^46^

### Study visit overview

The LEAN trial involved eight patient-related visits at their nearest trial site. The study was divided into four stages: (1) screening, enrolment, randomisation and baseline investigations (visits 1 and 2, over a maximum period of 14 days), (2) 336 days of study treatment (visits 3–6, over 48 weeks), (3) primary endpoint assessment including liver biopsy (visit 7, within 1 day of the EOT) and (4) post-treatment follow-up assessment (visit 8, 12 weeks after EOT). If the trial investigating team or the trial participant suspected an AE, an unscheduled visit was arranged within 24 h.

The schedule for the study visits and data collection is summarised in table 1. All participants were asked to attend each visit under fasting from eating or drinking (with exception of water) for a minimum of 8 h prior to each visit. A follow-up liver biopsy (ie, primary endpoint) was obtained under ultrasound-guidance after completion of the 48-week study treatment. Wherever possible, a 16-gauge biopsy needle and a specimen length of a minimum of 15 mm were preferred. The liver tissue was prepared at the local trial sites in preparation for histological assessment (under light microscopy) at the central trial site at the Queen Elizabeth University Hospital Birmingham. On receipt, the two central ‘blinded’ central histopathologists recorded the size and quality of the histology slides. A minimum of four unstained slides were available for each liver biopsy to enable repeat staining (H&E, haematoxylin van Gieson, Ubiquitin) to ensure adequate quality for interpretation.

**Table 1:** Trial schedule of data collection

	Screening		Treatment (TD, treatment day)					Follow-up
	Visit 1 (Max -14 days to TD1)	Visit 2 (1 day prior to TD1)	Visit 3 (TD 28)	Visit 4 (TD 84)	Visit 5 (TD 168)	Visit 6 (TD 252)	Visit 7 (1 Day + TD 336/ End of Treatment (EOT))	Visit 8 (12 weeks after EOT)
Informed consent	**X**							
Clinical assessment *	**X**		**X**	**X**	**X**	**X**	**X**	**X**
Vital signs†	**X**		**X**	**X**	**X**	**X**	**X**	**X**
ECG/urine dipstix	**X**			**X**	**X**	**X**	**X**	**X**
Standard blood tests‡	**X**		**X**	**X**	**X**	**X**	**X**	**X**
Screening blood tests§	**X**							
Lipid profile serum insulin	**X**			**X**	**X**		**X**	**X**
OGTT (non-diabetics only)	**X**						**X**	
Non-invasive fibrosis markers¶	**X**						**X**	**X**
Metabolic substudies**		**X**		**X**				
Questionnaires††	**X**						**X**	**X**
**Liver biopsy**	−‡‡						**X**	
Adverse/clinical events §§			**X**	**X**	**X**	**X**	**X**	**X**
Study medication dispensed		**X**¶¶	**X**	**X**	**X**	**X**		

*Clinical assessment: complete history/examination (visit 1), focused history/examination (visits 2–8).

†Vital signs: HR, blood pressure, weight, height, waist:hip circumference, body temperature, SaO_2_, RR.

‡Standard fasting blood tests: full blood count, U+E, liver function tests, international normalised ratio, thyroid function tests, glucose and HbA1c (*except visit 3*).

§Screening blood tests: HBsAg, HCV Ab, anti-mitochondrial antibody /ASA/immunoglobulins, ferritin/transferrin saturation, caeruloplasmin, α1AT, α-feta protein (AFP).

¶FibroMAX panel (FibroTest, SteatoTest, NashTest), enhanced liver fibrosis tests and transient elastography (Fibroscan; optional depending on availability).

**Optional metabolic substudy: two-step hyperinsulinaemic euglycaemic clamp with stable isotope studies and adipose microdialysis.

††Questionnaires: AUDIT, Block Brief 2000 Food Frequency Questionnaire, HR-quality of life (SF-36v2).

‡‡Diagnostic liver biopsy performed as part of standard National Health Service care ≤6 months of screening visit 1. Two independent liver histopathologists will review the liver biopsy to assess whether the patients meets the histological inclusion criteria.

§§Adverse events/bloods and clinical events will be monitored continuously until completion of follow-up and 30 days after. Calcitonin and AFP levels will be measured at visits 1, 5, 7 and 8.

¶¶If the study patient meets the eligibility criteria, he/she will be randomised at visit 2 to receive liraglutide (Victoza) or placebo. The allocated blinded study treatment will be dispensed at visit.

### Storage of trial samples

Liver biopsy tissue specimens were collected, paraffin-fixed and stored at the diagnostic archive of the department of cellular pathology (University Hospital Birmingham). Serum and plasma samples collected at visit 1 (screening), visits 4, 5, 7 (EOT) and 8 (12 weeks post-EOT) were stored frozen in 0.5–1.0 mL aliquots at –80°C at the Institute of Biomedical Research, University of Birmingham. Where possible, additional blood (buffer coat) was obtained at visits 1 and 7 for future DNA extraction and stored at –80°C. Both specimen storage banks hold a license from the Human Tissue Authority to store tissue for research purposes.

### Treatment compliance

Treatment compliance was monitored by a review of the used prefilled treatment pens, participant injection sites and the participant’s self-filled ‘standardised treatment and clinical events booklet’ at each study visit. The latter provided written evidence of dosage, time and date when each patient administered the study drug.

### Data handling, quality assurance, record keeping and retention

Data management was undertaken according to the standard operating procedures of the CRCTU at the University of Birmingham, UK. The CRCTU was fully compliant with the Data Protection Act 1998 and the International Conference on Harmonisation Good Clinical Practice (ICH GCP). The CRCTU was responsible for monitoring the trial and providing annual reports to the MHRA. The trial was registered with the Data Protection Act website at the University of Birmingham. Participant identifiable data were shared only within the clinical team on a need-to-know basis to provide clinical care, and to ensure good and appropriate follow-up. Patient identifiable data were also shared with approved auditors from the NRES, competent authorities (including MHRA, European Medicines Agency (EMA) and Food and Drug Administration (FDA)), sponsor (University of Birmingham), NHS R&D departments and the primary care practitioner. All LEAN participants provided specific written consent at trial entry to enable data to be shared with the above. Otherwise, confidentiality was maintained throughout the trial and thereafter. On completion of the trial, data will be transferred to a secure archiving facility at the University of Birmingham, where data will be held for a minimum of 10 years and then destroyed.

### Case report forms

CRFs included baseline/follow-up medical history and physical examinations to capture comorbidities and concomitant medications in the trial’s electronic database. Other CRFs incorporated in the electronic database included: laboratory tests and questionnaire results were recorded for visit 1 (eligibility criteria) through to visit 8; safety monitoring during the treatment follow-up periods; central site histopathology reports of liver biopsy specimens; specialist non-invasive markers of liver disease; AE reporting and study drug dispensing forms for study treatment adherence and accountability.

### Sponsorship, indemnity and monitoring

The University of Birmingham acted as the sponsor of the trial. As sponsor the University was responsible for the general conduct of the study and indemnified the trial centre against any claims, arising from any negligent act or omission by the University in fulfilling the sponsor role in respect to the study. Both on-site and off-site monitoring of the trial were performed as per the LEAN Trial Quality Management Plan.

### Sources of funding

The trial was funded by the Wellcome Trust (Clinical Research Fellowship awarded to MJA, 200), Novo Nordisk Ltd (free study drug supply, educational grant) and the NIHR liver BRU.

## Trial status

Recruitment into the LEAN trial started in August 2010 and ended in May 2013, with 52 patients (104% of target enrolment) randomised from five trial sites (Birmingham 31; Nottingham 12; Hull 6; Leeds 3; Southampton 0). This number is 2 more than planned so as to allow all participants that had registered/consented and found to be eligible to participate in the trial. Online supplementary figure S2 summarises the recruitment rate throughout the trial. A total of 73 patients were registered for the trial, 21 (29%) of whom were not eligible or withdrew consent before randomisation to the trial. Failure to meet the histological inclusion criteria (after central histopathology review) was the most frequent reason for ineligibility. The treatment follow-up of LEAN participants is currently ongoing and the last trial visit of the last participant is due to take place in July 2014.

## Discussion

Compliance with the trial protocol and safety profile of liraglutide was reviewed on a biannual basis by an independent DMC, and no concerns were raised.

### Challenges in trial design

Despite recent advances in non-invasive markers of liver injury (eg, transient elastography, serum fibrosis markers), liver biopsy remains the recommended method for the assessment of disease activity for phase II/III trials.^33^ Liver biopsy is not without its limitations (such as sampling error, invasive nature and patient reluctance for repeat sampling^47^), but until the accuracy of serial measurements of non-invasive markers have been formally validated, it will be required for trials in NASH. The LEAN trial has attempted to minimise these limitations. First, liver biopsies (<6 months of screening) performed for routine NHS diagnostic purposes were incorporated into the eligibility criteria and utilised as the baseline comparator, rather than performing two biopsies for the sole purpose of the trial. This approach is widely accepted in trials of NASH. Second, all of the liver biopsies (baseline, primary endpoint) underwent a blinded central review by two independent expert liver histopathologists (RMB and SGH) at the one site, ensuring that only patients with ‘definite’ NASH were recruited to the trial and reducing intra/interassessor variability, which has previously been reported between trial sites.^48^

In 2011, Sanyal and colleagues (update from AASLD research workshop, 2009) published expert guidance on clinical trial design in patients with NASH.^33^ Even though the LEAN trial design preceded this workshop, the definition of NASH and the outcome measures were in keeping with their recommendations. Patients with NASH have a higher risk of liver-related mortality than those with simple hepatic steatosis (± mild inflammation).^49^ ^50^ Owing to the long time-span of NASH progression (ie, 10–20 years) to end-stage liver failure/death it is impractical to perform therapeutic trials with mortality as the primary outcome measure. Therefore, we elected to use the disappearance of NASH with no worsening of fibrosis as ‘surrogate’ primary endpoint in LEAN. With this in mind, a 48-week treatment duration was selected, rather than 2–5 years, which would be required if we were aiming to demonstrate significant improvements in fibrosis. NAS has been incorporated as a secondary outcome measure (including the individual components of NAS) to represent disease activity,^31^ rather than as the primary outcome as previously reported.^48^ ^51^ NAS alone was not originally designed to infer absence or presence of NASH,^52^ which we deemed a more meaningful clinical outcome.

We elected to recruit patients with and without T2D to enhance recruitment rates and broaden the safety data in liraglutide in NASH, but under the provision that patients with diabetes must have moderate glycaemic control (HbA1c <9%) on diet ± oral hypoglycaemic medications (with the exception of TZDs and other potential confounders, ie, GLP-1 based therapy) prior to trial entry. With the knowledge that diabetes is a potential confounding factor, randomisation was programmed to stratify for diabetes to ensure equal numbers in each treatment arm.

The efficient recruitment for clinical trials in NASH remains a challenge, mainly due to the requirement for liver biopsy, which has been compounded by the recent uptake of non-invasive markers (eg, transient elastography) in the UK resulting in a decline in liver biopsy requests in some recruiting centres.^37^

### Safety profile of liraglutide

Prior to the start of the LEAN trial, the summary of product characteristics (SmPc) for liraglutide (Victoza) stated special warnings and precautions for use in moderate/severe renal impairment, moderate/severe congestive heart failure (NHYA class III/IV), pre-existing thyroid disease and in patients at risk of pancreatitis/pancreatic carcinoma.^53^ In turn, the eligibility criteria (box 1) reflected these warnings by excluding patients with or at risk of such conditions. In particular, based on the preclinical incidence of thyroid C-cell tumours in rodent models and the manufacturer’s ‘black box’ warning in humans,^53^ all patients with a personal/family history of thyroid carcinoma, multiple endocrine neoplasia syndrome type 2 and/or abnormal thyroid examination (goiter, nodules) were excluded from the trial. In addition, serum calcitonin, thyroid function tests and clinical thyroid examination were monitored throughout the trial as a precautionary measure.

In keeping with both the US FDA^54^ and EMA^55^ recommendations, all patients in LEAN were given written/verbal advise about the risks and carefully monitored for signs and symptoms indicative of pancreatitis. In March 2013, a small study (n=8) by Butler *et al*^56^ reported pancreatic cellular changes, consistent with pancreatic duct metaplasia, in organ donors who had received GLP-1 therapy for diabetes prior to death. In response in July 2013, the EMA's committee of Medicinal Products for Human Use (CHMP) critically appraised the study and all other non-clinical/clinical data available, and concluded that the current evidence did not confirm an increased risk of pancreatic AEs with GLP-1 based therapies.^57^ Subsequently, the current safety measures adopted by the LEAN trial will continue until further information is made available.

### Summary

To the best of our knowledge, the LEAN trial is the first multicentre, double-blinded, placebo-controlled RCT designed to investigate whether the long-acting GLP-1 analogue, liraglutide, is safe and improves liver histology in overweight patients with NASH. The enrolment of the required sample size was completed in May 2013 and the final results are expected by the end of 2014. The full LEAN protocol (V.7.0) can be obtained from the NIHR liver biomedical research unit and CRCTU at the University of Birmingham (LEAN@trials.bham.ac.uk).

## Supplementary Material

Author's manuscript
