# Supplementary material for: Liraglutide efficacy and action in non-alcoholic steatohepatitis (LEAN): study protocol for a phase II multicentre, double-blinded, randomised, controlled trial
Source: BMJ Open. 2013 Nov 4;3(11):e003995. doi: 10.1136/bmjopen-2013-003995 (PMC3822302; doi:10.1136/bmjopen-2013-003995)
Supplement: Author's manuscript [file bmjopen-2013-003995.draft_revisions.pdf]

Liraglutide Efficacy and Action in Non-alcoholic Steatohepatitis (LEAN): Study protocol for a Phase II multi-centre, double-blinded randomised-controlled trial

Journal:	*BMJ Open*
Manuscript ID:	bmjopen-2013-003995
Article Type:	Protocol
Date Submitted by the Author:	11-Sep-2013

Complete List of Authors:	Armstrong, Matthew; University of Birmingham, NIHR Biomedical Research Unit and Centre for Liver Research<br>Barton, Darren; University of Birmingham, NIHR Liver BRU Clinical trials group (EDD), CRUK clinical trials unit<br>Gaunt, Piers; University of Birmingham, NIHR Liver BRU Clinical trials group (EDD), CRUK clinical trials unit<br>Hull, Diana; University of Birmingham, NIHR Liver BRU and Centre for Liver Research<br>Guo, Kathy; University of Birmingham, NIHR Liver BRU and Centre for Liver Research<br>Stocken, Deborah; Newcastle University, Newcastle Clinical Trial Unit, Institute of Health and Society,<br>Gough, Professor Stephen; University of Oxford,<br>Tomlinson, Jeremy; University of Birmingham, Centre for Endocrinology, Diabetes and Metabolism<br>Brown, Rachel; University Hospital Birmingham, Department of Cellular Pathology<br>Hubscher, Stefan; University Hospital Birmingham, Department of Cellular Pathology<br>Newsome, Philip; University of Birmingham, NIHR Liver BRU and Centre for Liver Research

**Primary Subject Heading**:	Gastroenterology and hepatology
Secondary Subject Heading:	Pharmacology and therapeutics, Research methods, Pathology
Keywords:	CLINICAL PHARMACOLOGY, Hepatobiliary disease < GASTROENTEROLOGY, HISTOPATHOLOGY, Hepatology < INTERNAL MEDICINE

SCHOLARONE™ Manuscripts

Liraglutide Efficacy and Action in Non-alcoholic Steatohepatitis (LEAN): Study protocol for a Phase II multi-centre, double-blinded randomised-controlled trial

Matthew J. Armstrong[1,2*], Darren Barton[3], Piers Gaunt[3], Diana Hull[1], Kathy Guo[1], Deborah Stocken[4], Stephen CL. Gough[5], Jeremy W. Tomlinson[6], Rachel M. Brown[7], Stefan G. Hübscher[7,8], Philip N. Newsome[1,2*]; on behalf of the **LEAN trial team**

1. NIHR Liver BRU and Centre for Liver Research, University of Birmingham, Birmingham, UK, B15 2TT.

2. Liver and Hepatobiliary Unit, Queen Elizabeth Hospital Birmingham, Birmingham, UK, B15 2WB

3. NIHR Liver BRU Clinical trials group (EDD), CRUK clinical trials unit, University of Birmingham, Birmingham, UK B15 2TT.

4. Newcastle Clinical Trial Unit, Institute of Health and Society, Baddiley-Clark Building, Newcastle University, Richardson Road, Newcastle upon Tyne, NE2 4AX.

5. Oxford Centre for Diabetes, Endocrinology and Metabolism, University of Oxford, Churchill Hospital, Oxford, OX3 7LJ

6. Centre for Diabetes, Endocrinology and Metabolism, University of Birmingham, Birmingham, UK, B15 2TT.

7. Department of Cellular Pathology, Queen Elizabeth Hospital Birmingham, Birmingham, UK, B15 2WB

8. School of Cancer Sciences, University of Birmingham, Birmingham, UK, B15 2TT

For peer review only

For peer review only - http://bmjopen.bmj.com/site/about/guidelines.xhtml

*Corresponding authors:

Dr Matthew J. Armstrong, Wellcome Trust Clinical Research Fellow, NIHR Biomedical Research Unit and Centre for Liver Research, University of Birmingham, Birmingham, B15 2TT. Email: mattyarm2010@googlemail.com. Tel: 07968470622.

Professor Philip N. Newsome, Professor of Hepatology, NIHR Biomedical Research Unit and Centre for Liver Research, University of Birmingham, Birmingham, B15 2TT. Email: p.n.newsome@bham.ac.uk Tel: 0121 414 5614.

Email addresses of co-authors:

Darren Barton: d.barton@bham.ac.uk

Piers Gaunt: p.gaunt@bham.ac.uk

Diana Hull: d.hull@bham.ac.uk

Kathy Guo: k.guo@bham.ac.uk

Deborah Stocken: Deborah.stocken@newcastle.ac.uk

Professor Stephen Gough: stephen.gough@ocdem.ox.ac.uk

Dr Jeremy Tomlinson: j.w.tomlinson@bham.ac.uk

Dr Rachel Brown: Rachel.Brown@uhb.nhs.uk

Prof Stefan Hübscher: s.g.hubscher@bham.ac.uk

Figures: 2

Tables: 2

For peer review only

ABSTRACT (300 words):

Introduction: Non-alcoholic steatohepatitis (NASH) is now the commonest cause of chronic liver disease. Despite this, there are no universally accepted pharmacological therapies for NASH. Liraglutide (Victoza®), a human glucagon-like peptide-1 analogue, has been shown to improve weight loss, glycaemic control and liver enzymes in type 2 diabetes. There is currently a lack of prospective-controlled study investigating the efficacy of GLP-1 analogues in patients with NASH.

Methods and analysis: LEAN is phase II, multi-centre, double-blinded, placebo-controlled, randomised clinical trial designed to investigate whether 48 weeks treatment with 1.8mg liraglutide will result in improvements in liver histology in patients with NASH. Adult, overweight (body mass index ≥25kg/m^2) patients with biopsy-confirmed NASH were assessed for eligibility at 5 recruitment centres in the UK. Patients who satisfied the eligibility criteria were randomly assigned (1:1) to receive once-daily subcutaneous injections of either 1.8mg liraglutide or liraglutide-placebo (control). Using A'Hern's single stage phase II methodology (significance level 0.05; power 0.90) and accounting for an estimated 20% withdrawal rate, a minimum of 25 patients were randomised to each treatment group. The primary outcome measure will be centrally assessed using an intention-to-treat analysis of the proportion of evaluable patients achieving an improvement in liver histology between liver biopsies at baseline and after 48 weeks of treatment. Histological improvement will be defined as a combination of the disappearance of active NASH and no worsening in fibrosis.

Ethics and dissemination: The protocol was approved by the National Research Ethics Service (East Midlands – Northampton committee; 10/H0402/32) and the

For peer review only - http://bmjopen.bmj.com/site/about/guidelines.xhtml

MHRA. Recruitment into the LEAN started in August 2010 and ended in May 2013, with 52 patients randomised. The treatment follow-up of LEAN participants is currently ongoing and is due to finish in July 2014. The findings of this trial will be disseminated through peer-reviewed publications and international presentations.

Trial registration: clinicaltrials.gov NCT01237119.

KEYWORDS: Non-alcoholic fatty liver, glucagon-like peptide 1, hepatocyte ballooning, therapy, safety.

ABBREVIATIONS: A1AT, alpha-1 anti-trypsin; AFP, alpha-feta protein; ALT, alanine transaminase; AMA, anti-mitochondrial antibody; ASMA, anti-smooth muscle antibody; AUDIT, Alcohol Use Disorders Identification Test; BMI, body mass index; CK-18, cytokeratin-18; CRP, c-reactive protein; DMC, data management committee; DPP-IV, dipeptidyl peptidase IV; ELF, enhanced liver fibrosis test; FBC, Full blood count; FFQ, food frequency questionnaire; GLP-1, glucagon-like peptide-1; HbA1c, glycosylated haemoglobin; HBVsAg, hepatitis B virus surface antigen; HCVab, hepatitis C virus antibody; H&E, haematoxylin and eosin; HOMA-IR, homeostatic model assessment of insulin resistance; INR, international normalised ratio; LEAD, Liraglutide Effect and Action in Diabetes; LEAN, Liraglutide Efficacy and Action in NASH; LFTs, liver function tests; LSE, liver stiffness evaluation; MHRA, Medicines and Healthcare Products Regulatory Agency; NAFLD, Non-alcoholic fatty liver disease; NASH, Non-alcoholic steatohepatitis; NAS, NAFLD activity score; NHANES, National Health and Nutrition Examination Survey; NRES, National Research Ethics Service; OD, once-daily; OGTT, oral glucose tolerance test; RCT, randomised-controlled trial;

For peer review only - http://bmjopen.bmj.com/site/about/guidelines.xhtml

For peer review only

R&D, Research and Development; SAE, serious adverse event; SUSAR, suspected unexpected serious adverse reaction; TFTs, thyroid function tests; TMG, trial management group; TSH, thyroid stimulating hormone; TZD, thiazolidinedione.

For peer review only

For peer review only - http://bmjopen.bmj.com/site/about/guidelines.xhtml

1.1 Introduction

Non-alcoholic fatty liver disease (NAFLD) is now the commonest cause of chronic liver disease, affecting up to 30% of the general population (1-3) and 70-90% of high-risk individuals (3, 4). This prevalence relates to the dramatic rise in recent years of morbid obesity and type 2 diabetes. Even though simple hepatic steatosis (without fibrosis) is arguably a benign condition, up to a quarter of patients with NAFLD have the more severe, inflammatory condition known as non-alcoholic steatohepatitis (NASH) (5). Patients with NASH have an increased risk of progression to cirrhosis, liver failure and hepatocellular carcinoma (6), and are expected to become the commonest indication for liver transplantation in forthcoming years (7). Despite this, there are no universally accepted pharmacological therapies for NASH. Therefore the need for novel, safe agents in NASH is of paramount importance to prevent disease progression and the accompanying clinical burden.

The strong association of NASH with the metabolic syndrome, in particular central adiposity and insulin resistance, provides strong rationale for investigating therapies that induce weight loss and insulin sensitivity. The gut-derived incretin hormone, glucagon-like peptide-1 (GLP-1), is therefore an attractive target option in NASH. Native GLP-1 has a potent blood glucose-lowering action mediated via its ability to induce insulin secretion and reduce glucagon secretion in a glucose-dependent manner, as well as suppressing appetite and slowing gastric emptying (8). Human GLP-1, however, only has a short half-life (1.5-2.0 mins) as it is rapidly degraded by the enzyme dipeptidyl peptidase-4 (9). Liraglutide (Victoza®) is a long-acting (half-

For peer review only - http://bmjopen.bmj.com/site/about/guidelines.xhtml

life 13 hours) GLP-1 analogue with 97% structural homology to the native hormone and is administered once daily (OD) by subcutaneous injection (10). Liraglutide has been shown to cause dose-dependent weight loss (11, 12), decrease glycosylated haemoglobin (HbA1c), systolic blood pressure and improve beta-cell function (13-18). Subsequently, it has been licensed for glycaemic control in overweight patients with type diabetes (19). There is, however, a paucity of data in patients with liver disease, and in particular histological-defined NASH.

GLP-1 analogues, including liraglutide, have been shown to improve liver enzymes, oxidative stress and hepatic steatosis in murine models *in vivo* and in isolated *in vitro* murine and human hepatocyte studies (20-25). To date, human studies investigating the effect on liver injury have been limited to case reports (26, 27), solitary case series (n=8) (28) and retrospective (*liver enzyme*) studies in patients with type 2 diabetes (29). A large meta-analysis of six phase III randomized-controlled trials (RCT), that comprised the LEAD (Liraglutide Effect and Action in Diabetes) program (>4000 patients), highlighted that 26-weeks treatment with 1.8mg OD liraglutide was well-tolerated and resulted in significant improvements in liver enzymes compared to placebo-control in overweight patients with type diabetes (30). However, limitations of this study were the retrospective nature of its analysis and the lack of any liver biopsy data.

On this basis, we hypothesised that 48 weeks treatment with liraglutide would result in significant improvements in liver histology in overweight patients with NASH. To

For peer review only - http://bmjopen.bmj.com/site/about/guidelines.xhtml

test this hypothesis, we designed a phase II, multi-centre, double-blinded, placebo-controlled RCT, entitled ‘Liraglutide Efficacy and Action in NASH (LEAN).’

For peer review only

For peer review only - http://bmjopen.bmj.com/site/about/guidelines.xhtml

1.2 Methods

1.2.1 Study Design Overview

LEAN is a 48 week multi-centre, double-blinded, placebo-controlled randomised clinical trial of treatment with the once daily human GLP-1 analogue, liraglutide (Victoza®), for adults with biopsy-proven NASH. Screening was undertaken within 14 days of randomisation to assess eligibility and collect baseline data. Patients who satisfied the eligibility criteria were randomly assigned (1:1) to receive OD subcutaneous injections of either 1.8 mg liraglutide (experimental) or liraglutide-placebo (control). After which, a 12-week washout period is scheduled.

The primary outcome measure will be assessed using an intention-to-treat analysis of the proportion of evaluable patients achieving an improvement in liver histology between liver biopsies at baseline (within 6 months of screening) and after 48 weeks of treatment. Histological improvement will be defined as a combination of the disappearance of active steatohepatitis (i.e. disappearance of hepatocyte ballooning) and no worsening in fibrosis (Kleiner Fibrosis score (31)). A schematic of the trial design is summarised in **Figure 1**.

1.2.2 Ethical and regulatory approval

The National Research Ethics Service (NRES) East Midlands – Northampton committee (previously known as Leicestershire, Northamptonshire and Rutland

For peer review only - http://bmjopen.bmj.com/site/about/guidelines.xhtml

Research Ethics Committee) (UK) and the Medicines and Healthcare products Regulatory Agency (MHRA) approved all versions (inc. current version 7.0) of the study protocol. In addition, all 5 recruitment sites obtained approval from their respective hospital Research and Development (R&D) departments prior to commencing screening.

1.2.3 Treatment groups

Patients who satisfied the eligibility criteria were randomly assigned on a 1:1 basis to 48-weeks treatment of either liraglutide (Victoza®; 1.8mg OD) or liraglutide-placebo control (1.8mg OD).

1.2.3.1 Liraglutide (active experimental group)

Liraglutide (Victoza®, Novo Nordisk A/S, Bagsvaerd, Denmark) was supplied in a cartridge contained in a pre-filled multi-dose disposable pen. Each pre-filled pen contained 18 mg liraglutide in 3 ml of clear, colourless, isotonic solution (including water for injections, disodium phosphate dehydrate, propylene glycol and phenol). Liraglutide was administered OD, at any time of the day, as a single subcutaneous injection into the abdomen, thigh or upper arm using the pre-filled pen (30 or 31 gauge needles). Participants were encouraged to inject liraglutide at the same time each day, according to which was the most convenient time for them. Participants were instructed to perform an air shot of 0.2 µl before the first use of each new pre-filled pen to ensure that it functioned correctly.

For peer review only - http://bmjopen.bmj.com/site/about/guidelines.xhtml

To improve gastro-intestinal tolerability participants underwent a 14-day dose titration period in keeping with previous reports (13-18). The dose was titrated by 0.6 mg every 7 days from a starting dose of 0.6mg OD until the maximum dose of 1.8 mg OD was achieved. Prior to the current trial design, no studies had investigated any form of GLP-1 based therapy in patients with biopsy-confirmed NASH or any other form of liver disease. Therefore, the rationale for using a dose of 1.8mg OD was based upon previous reports in overweight patients with or without type 2 diabetes (13-18). Furthermore, a large meta-analysis of six phase III clinical trials (LEAD program) of liraglutide therapy for poorly controlled type 2 diabetes found that patients with abnormal liver transaminases had a similar drug safety profile to those with normal liver transaminases. In addition, greater improvements in liver transaminases and CT-measured hepatic steatosis were seen with 1.8mg liraglutide than 1.2 and 0.6mg doses (30).

1.2.3.2 Liraglutide-placebo (inactive, placebo-control group)

Liraglutide-placebo (Victoza®, Novo Nordisk A/S, Bagsvaerd, Denmark) was packaged, administered and dose-titrated in an identical manner to the liraglutide comparator, described above. The composition of the placebo solution for injection was identical to its comparator, with the exclusion of the active liraglutide substance. A placebo was used to provide an assessment of the level of response with an injectable placebo, which could potentially be higher than that seen with oral placebo agents.

For peer review only - http://bmjopen.bmj.com/site/about/guidelines.xhtml

1.2.3.3 Concomitant Therapy

No dose reductions of liraglutide or placebo were allowed throughout the 48 week treatment period. Previous treatment with oral anti-diabetic drugs (metformin and/or sulphonylurea) was continued at the same dose in participants with type 2 diabetes at randomisation. In the event of recurrent major hypoglycaemic episodes (requiring medical or hospital intervention), the dose of the sulphonylurea was reduced by 50% at the discretion of the investigators. The reported rate of hypoglycaemia in the literature, with liraglutide monotherapy or in combination with metformin, is very low (13-18). However in the event of recurrent major hypoglycaemic episodes in which no dose reduction could be undertaken (i.e. not on a sulphonylurea) the subject was withdrawn from treatment at the discretion of the chief investigator.

Glycaemic control was assessed at each 12-weekly trial visit with self-measured plasma glucose readings and HbA1c. In the event that glycaemic control deteriorated, defined as HbA1c > 9.0% (75 mmol/mol), the subject was informed and counselled with regards to commencing open-labelled long-acting OD insulin detemir (Levemir®). However, the patient's participation in the trial was not jeopardised if they did not wish to start insulin detemir. The Insulin detemir dose was titrated by trial investigators in accordance with European guidelines (www.ema.europa.eu) to ensure that the subject's standard of diabetes care was not significantly compromised as a result of participating in the clinical trial. The HbA1c cut-off of >9.0% was based on the opinions of the TMG (MJA, PNN),

For peer review only - http://bmjopen.bmj.com/site/about/guidelines.xhtml

consisting of expert endocrinologists (SG, JWT), and in accordance with previous clinical trial guidance (32).

In addition to study medications, participants continued to receive standard National Health Services (NHS) care recommendations concerning life-style modifications (i.e. exercise, weight loss and dietary modification) and management of various co-existing illnesses throughout the trial. Patients were asked to limit alcohol consumption to less than 20 mg/day for females (i.e. 14 units/week) and 30 mg/day for males (i.e. 21 units/week). These levels were consistent with the UK Departmental of Health recommended daily alcohol allowance (British Medical Association 1995). Participants were not allowed any new prescription or over-the-counter therapies (i.e. herbal remedies, milk thistle) that may improve or worsen NASH throughout the duration of the trial. Potential NASH therapies that were not allowed during the trial duration included thiazolidinediones (TZDs), dipeptidyl peptidase (DPP) IV inhibitors, other GLP-1 receptor agonists (e.g. exenatide), vitamin E and orlistat. Steroids (oral or intravenous), methotrexate and/or amiodarone were also not permitted based on their ability to promote hepatic steatosis.

1.2.4 Outcome Measures

1.2.4.1 Primary Outcome Measure

The primary outcome measure is the proportion of participants with a significant improvement in liver histology between liver biopsies at baseline (i.e. within 6

For peer review only - http://bmjopen.bmj.com/site/about/guidelines.xhtml

months of screening) and at the end of 48-weeks treatment. The definition of a significant histological improvement requires both the disappearance of steatohepatitis (defined as a disappearance of hepatocyte ballooning) and no worsening of fibrosis, as assessed by the Kleiner scoring system (31). Hepatocyte ballooning is now widely recognised as the key lesion for distinguishing NASH from simple steatosis.

1.2.4.2 Secondary Outcome Measures

Secondary outcome measures include changes in; (a) overall NAFLD Activity Score (NAS) (31); (b) individual histological components of NAS, including lobular inflammation, steatosis, hepatocyte ballooning, and fibrosis; (c) serum markers of steatosis (SteatoTest™), NASH (NashTest™, caspase-cleaved cytokeratin-18 [CK-18 M30]), and fibrosis (Enhanced Liver Fibrosis (ELF; iQUR Ltd), FibroTest™); (d) Liver stiffness evaluation (LSE) with Transient Elastography (Fibroscan®, Echosens, Paris, France); (e) Insulin resistance (HOMA-IR); (f) Anthropometric measures including body weight, body mass index (BMI) and waist circumference; (g) Lipid profile and glycaemic control (HbA1c, fasting plasma glucose); (h) serum ALT levels; and (i) health-related quality of life (SF-26 version 2.0) and nutrition (Block Brief 2000 Food Frequency Questionnaire questionnaires).

For peer review only - http://bmjopen.bmj.com/site/about/guidelines.xhtml

1.2.5 Analytical Methods

1.2.5.1 Liver Histopathology

Two independent liver histopathologists (SGH, RB) at the central trial site (Birmingham, UK) will perform all the histopathological assessments using an in-house designed proforma (**Supplementary table 1**). Both histopathologists will be blinded to the clinical, laboratory and study treatment allocation. The histological diagnosis of NASH will be established using haematoxylin and eosin (H&E) staining and haematoxylin van Gieson stains of formalin fixed paraffin-embedded liver tissue. Both the baseline and end of treatment (48 weeks) biopsies will be reported as either 'definite NASH,' 'uncertain NASH,' or 'not NASH.' The histological diagnosis of 'definite NASH' is defined as a combination of >5% macrovesicular steatosis, hepatocyte ballooning (+/- Mallory's Hyaline) and lobular inflammation (mixed infiltrate, related to foci of ballooning) (33). The assessment of ballooning is subjective, and thus for 'uncertain' hepatocyte ballooning, a key component of the diagnosis of NASH, ubiquitin immunohistochemistry will be used to identify material compatible with Mallory's hyaline (**Figure 2**). To validate the quality of the biopsy specimen the core specimen length will be measured and the number of portal tracts will be recorded.

The NAS will be calculated based on the Kleiner classification (31). The NAS is score out of 8, with 8 representing the highest activity. The NAS is the sum of scores of the three components of the histological scoring system, namely steatosis (0 = < 5%, 1 =

For peer review only - http://bmjopen.bmj.com/site/about/guidelines.xhtml

5-33%, 2 = >33-66%, 3 = >66%), lobular inflammation (0 = no foci, 1 = <2 foci/200x, 2 = 2-4 foci/200x, 3 = >4 foci) and hepatocyte ballooning (0= none, 1 = few ballooned cells, 2 = many cells/prominent ballooning). The Kleiner scoring system for NAFLD fibrosis (F0-F4) (31) and a modified version of the Ishak score (34) (F0-F6) (**Supplementary table 1**) will be used to evaluate the stage of fibrosis in each biopsy specimen. The Ishak score was modified from the original scoring system, reported in 1995 (34), in order to include the zone 3 peri-cellular/peri-sinusoidal fibrosis, which is characteristically seen in NASH. Portal tract changes (inflammation, interface hepatitis, ductular reaction), an intrinsic feature of NASH, will also be recorded (35).

The pathologists will assess the biopsies independently and fill in separate forms. Cases where there is disagreement on the classification, as 'NASH' or 'not NASH,' will be reviewed and a consensus opinion given. Also discrepancies of more than 1 point on any of the scoring scales (NAS, Kleiner fibrosis scoring system and modified Ishak score) will be reviewed and an amended consensus view offered. Discrepancies of only 1 point will not be altered.

1.2.5.2 Clinical and Laboratory data

Fasting blood samples will be analysed for full blood count, urea, creatinine and electrolytes, thyroid stimulating hormone (TSH), lipid profile (total cholesterol, HDL, triglycerides), liver function tests (LFT), prothrombin time, International Normalised Ratio (INR), amylase, alpha-feta protein (AFP), c-reactive protein (CRP), glycosylated

For peer review only - http://bmjopen.bmj.com/site/about/guidelines.xhtml

haemoglobin (HbA1c), calcitonin and plasma glucose using standard laboratory methods (Roche Modular system, Roche Ltd, Lewes, UK). Serum Insulin (Mercodia, Uppsala, Sweden), non-esterified fatty acids (NEFA) (Zen-Bio, Research Triangle Park, NC, USA) and CK-18 M30 (M30 Apoptosense ELISA Kit; PEVIVA AB, Bromma, Sweden) will be measured in-house using commercially available colorimetric ELISAs. Serum caspase-cleaved cytokeratin-18 (CK-18 M30) and the Enhanced Liver Fibrosis (ELF) Test were performed at study entry to assess hepatic apoptosis and fibrosis, respectively. The FibroMax™ panel (consisting of the SteatoTest™, NashTest™, FibroTest™) will be undertaken by Lab 21 Ltd (Cambridge, UK). The ELF test, which combines three direct serum markers of fibrosis (hyaluronic acid, pro-collagen III amino terminal peptide and tissue inhibitor of metalloproteinase 1) using an algorithm developed by the European Liver Fibrosis Group (36), will be performed on fasting serum stored at -80 degrees by a commercial laboratory (iQUR Ltd, Royal Free Hospital, London, UK).

Type 2 diabetes was considered present if patients had a recorded diagnosis in their medical records or if the fasting plasma glucose was ≥ 7.0 mmol/L and/or if the 2-hour 75g oral glucose tolerance test (OGTT) plasma glucose was ≥ 11.1 mmol/L. All patients without a recorded history of T2D were screened with an OGTT. Impaired glucose tolerance was defined as a 2-hour plasma glucose between 7.8 and 11.1 mmol/L. HOMA-IR, a marker of insulin resistance, was calculated in the standard fashion: Glucose x Insulin ÷ 22.5.

For peer review only - http://bmjopen.bmj.com/site/about/guidelines.xhtml

Measurements of weight (kg), height, systolic/diastolic blood pressure and waist:hip circumferences were recorded. Waist and hip circumferences were defined as the circumferential measurements immediately above the level of the iliac crests and at the level of the greater trochanters, respectively. Body mass index (BMI) was defined as weight in kilograms divided by the square of the height in metres (kg/m^2).

Liver stiffness evaluation (LSE) was measured using Transient Elastography (Fibroscan®, Echosens, France). The median value and interquartile range (IQR) of 10 validated measurements were recorded within the range of 2.5 to 75 kPa. The XL probe was used on individuals who have a BMI greater than 30 kg/m^2 or when the Fibroscan® 502 Touch machine (automated) recommends its use over the M-probe. To achieve a valid LSE (median of successful liver stiffness measurements) the operator had to obtain all of the following 3 criteria: 1) ≥10 successful liver stiffness measurements; 2) IQR/median ratio <0.30; and 3) ≥60% measurement success rate (37).

1.2.5.3 Patient questionnaires

Quality of life (QOL) was assessed by the Short Form 36 version 2.0 (SF-36v2) health-related QOL questionnaire (QualityMetric Health Outcomes Solutions, Lincoln, USA). The SF-36v2 questionnaire was a practical, reliable and valid measure of physical and mental health that could be completed in 5-10 mins. It consisted of 36 questions that assessed the functional health and well-being from the study subject's point of view (38). The Block Brief 2000 Food Frequency Questionnaire (FFQ) (Block Dietary

For peer review only - http://bmjopen.bmj.com/site/about/guidelines.xhtml

Data Systems, California, US) was completed by each subject to assess usual and customary intake of a wide array of nutrients and food groups. The food list incorporated in the Block questionnaire was developed from the National Health and Nutrition Examination Survey (NHANES) III dietary recall data. The Block Brief 2000 FFQ consisted of a well-validated self-administered questionnaire consisting of 70 food related questions and took approximately 15 mins to complete (39). Pictures of standardized serving sizes and an American-to-English food translation sheet (i.e. 'Catsup' = tomato 'Ketchup') were used to aid completion of the questionnaire.

The Alcohol Use Disorder Identification Test (AUDIT) questionnaire was used to assess the frequency of alcohol consumption and screen out alcohol-related problems, and dependence symptoms (40). The AUDIT questionnaire consisted of a 10-item questionnaire that took 2-5 mins to complete. The questionnaire has a positive predictive value of 98% for hazardous drinking, and a negative predictive value of 97% for alcohol dependence. The overall score ranges from 0 to 40, with a score of less than 8 being a good indication of insignificant alcohol consumption.

All questionnaires were completed at baseline (visit 1), end of treatment (visit 7) and 12 weeks post treatment (visit 8). Analysis will report the change from baseline scores to both the end of treatment and follow up scores.

For peer review only

1.2.6 Statistical Justification and Outcome Analysis

1.2.6.1 Sample size Justification

This is an early phase II trial randomising patients equally between two treatment arms - one experimental (liraglutide) and one control (placebo). The primary aim is not to determine the efficacy of liraglutide compared to placebo but to assess whether the efficacy and safety profile of liraglutide is worthy of further investigation. Recruiting patients into a no treatment control group provides simultaneous unbiased assessment of comparable patient groups.

At the time of the study design there were no available data to estimate histological response with 48 weeks treatment of liraglutide (Victoza®). Based on previous non-GLP-1 pharmaceutical trials in NASH utilising improvements in liver histology as a primary end-point (41, 42), it was assumed that 14-17% of patients undergoing current standard of care (placebo) would have an improvement in NASH by week 48. It was estimated that 20% of the placebo-control arm would achieve an improvement in liver histology, based in part on the knowledge that the placebo-effect might be exaggerated by the subcutaneous injection route of administration (vs. oral route in previous NASH trials) in the current trial. To justify further investigation of liraglutide treatment, a clinically relevant improvement in liver histology was required in at least 50% of patients. The sample size was calculated using A'Hern's single stage phase II methodology (43), with a significance level of 0.05 (type 1 error) and power of 0.90 (type II error). The design required 21

For peer review only - http://bmjopen.bmj.com/site/about/guidelines.xhtml

evaluable patients in the treatment group. The published literature in NASH trials reported on average a participant withdrawal rate of 10-20% (41, 42, 44). Therefore, to account for a 20% withdrawal rate the recruitment target was inflated from 21 to 25 patients per treatment group; the total recruitment target being 50 patients randomised in a 1:1 allocation ratio to either Liraglutide or placebo.

2.5.2 Analysis of Outcome Measures

All evaluable patients will be analysed on the intention-to-treat principle. Evaluable patients are defined as those who have had an end of treatment biopsy (visit 7), irrespective of the amount of treatment they have received. Patients will be categorised as either achieving the primary histological end-point (i.e. disappearance in NASH) or not. The proportion of patients with a reported improvement in liver histology will be presented and compared across treatments descriptively with 95% confidence intervals. The proposed A'Herns design stipulates that 8 or more evaluable patients out of 21 in the experimental treatment group (liraglutide) need to achieve the defined improvement in liver histology for treatment with liraglutide to be deemed worthy of further investigation with a phase III trial. Analyses will be presented for the subgroups of patients with and without type 2 diabetes. Patients who have not had an end of treatment biopsy will be classed as non-evaluable and will not be included in the primary analysis.

Secondary analysis of the primary outcome measure will report (a) the numbers and proportion of patients that did not have an end of treatment biopsy and the reasons

For peer review only - http://bmjopen.bmj.com/site/about/guidelines.xhtml

for this (these will be classified as 'no histological improvement') and (b) the numbers and proportion of patients that were considered to have had sufficient treatment and an end of treatment biopsy. In addition, an analysis that directly compares the two proportions for the separate treatment arms will be performed using the Chi-squared test.

Secondary measures collected as continuous and categorical variables will be presented with 95% confidence intervals descriptively across treatments using medians and proportions, respectively. Secondary measures collected as longitudinal data (including quality of life data, scored as per the questionnaire specific scoring manuals) will be presented descriptively across treatment groups as changes over time. A summary of all adverse events experienced by patients in both arms will be reported.

For peer review only - http://bmjopen.bmj.com/site/about/guidelines.xhtml

For peer review only

1.3 Conduct of the trial

1.3.1 Patient Selection

Eligible adults (≥ 18 years old) were identified and recruited at the participating trial site centres starting in August 2010 and by May 2013, 52 patients were recruited. Participating UK trial centres included the liver units at the Queen Elizabeth University Hospital (Birmingham, from Aug 2010), Queens Medical Centre (Nottingham, from May 2011), Southampton General Hospital (Southampton, Sept 2011), Hull Royal Infirmary (Hull, Nov 2011) and St. James Hospital (Leeds, from May 2012). All trial participants gave informed written consent at the beginning of the screening visit prior to undergoing any tests and procedures needed to assess eligibility.

Eligibility for the trial was determined at screening visit 1 by standard blood tests, clinical history (including written-confirmation of drug history where necessary) and physical examination/observations to identify other illnesses or contraindications for participation (Trial schedule figure). In addition, after receiving formal training the patient's ability to understand and self-administer the subcutaneous injections using the pre-filled treatment pen was assessed by an experience nurse specialist at screening visit 2. Patients who satisfied the eligibility criteria for the 48 week treatment trial at the Birmingham site were given the option to participate in a metabolic mechanistic sub-study. The sub-study involved two overnight admissions (approximately 22 hours) to the Wellcome Trust Clinical Research Facility

For peer review only - http://bmjopen.bmj.com/site/about/guidelines.xhtml

(Birmingham) to undergo a 2-step hyperinsulinaemic euglycaemic clamp with stable isotopes and adipose microdialysis on visits 2 (pre-treatment) and visit 4 (12-weeks treatment). A detailed summary of the metabolic sub-study will be published separately. A patient's decision to partake or withdraw from the metabolic sub-study did not affect their participation in the main 48 week trial.

1.3.1.1 Inclusion Criteria

The trial entry criteria were based on a diagnosis of 'definite' NASH on liver biopsy obtained within 6 months of screening. Prior to randomisation, two independent liver histopathologists (SGH, RB) from the central trial site (University Hospital Birmingham, UK) reviewed all of the liver biopsies (internal and external trial sites) of the potential participants to assess whether a diagnosis of 'definite' NASH was present. A 'definite' diagnosis of NASH was defined if all of the following were present on biopsy: (i) macrovesicular steatosis (>5%); (ii) hepatocyte ballooning (+/- Mallory Hyaline); and (iii) Lobular inflammation (mixed infiltrate, related to foci of ballooning). The two independent histopathology case report forms (CRFs) were reviewed by a trial investigator (MJA) and in the event that the histopathologists disagreed with regards to the diagnosis of NASH (i.e. one judged 'uncertain' and the other 'definite') a combined histopathology assessment was undertaken to determine the patient's eligibility status. Only patients with 'definite' NASH (either on two independent reports or after joint review) were classified as eligible.

For peer review only - http://bmjopen.bmj.com/site/about/guidelines.xhtml

All participants had to be ≥18 to <70 years old and have a body mass index (BMI) ≥ 25 kg/m^2 at screening. Patients with Type II Diabetes Mellitus at screening had to have stable glycaemic control (HbA1c <9.0%) and be managed by either diet and/or a stable dose of metformin/sulphonylurea. Patients without a history of type 2 diabetes prior to the screening visit underwent an OGTT at screening to determine their glycaemic status and were labelled as 'non-diabetic' if one or more of the following was confirmed:

- Impaired fasting glucose (IFG), defined using the European Criteria between 6.1 and 6.9 mmol/L
- Impaired glucose tolerance (IGT), defined as two-hour plasma glucose levels between 7.8 and 11.0 mmol/L on the 75-g OGTT
- Normal Fasting Plasma Glucose (FPG) < 6.1 mmol/L and Normal 2-hour plasma glucose levels < 7.8 on the 75g OGTT.

1.3.1.2 Exclusion Criteria

A detailed summary of the exclusion criteria is provided in **Table 1**. In brief, patients with a history or current significant alcohol consumption, poor glycaemic control (HbA1c > 9.0%), Child's Pugh B or C cirrhosis or another liver disease aetiology were excluded. The latter was confirmed with a full liver aetiology screen (drug-induced, viral hepatitis B/C, autoimmune, and genetic) at the screening visit. Past and current alcohol consumption was ascertained by a detailed review of the patients past medical, social history and by a self-administered AUDIT questionnaire with reference pictures to remind subjects of drink equivalents. Concomitant use of drugs

For peer review only - http://bmjopen.bmj.com/site/about/guidelines.xhtml

reported to be inducers (methotrexate, amiodarone, steroids) or potential therapies for NASH (TZDs, vitamin E), or other known hepatotoxins were assessed during the screening visit (**Table 1**). In keeping with previous clinical trials assessing GLP-1 therapies, patients with a history of acute/chronic pancreatitis (of any cause), pancreatic and thyroid carcinoma, and/or a family history of medullary thyroid carcinoma were also excluded.

1.3.2 Randomisation

Subjects who met all the eligibility criteria and provided written informed consent were randomly assigned on a 1:1 basis to either of the two study treatments (liraglutide *vs.* placebo) using computer generated randomisation at the Cancer Research UK Clinical Trials Unit (CRCTU). The randomisation was stratified to ensure that there were equal numbers of patients with/without type 2 diabetes in each treatment group and that each trial site had equal numbers of patients on each treatment. Trial subjects were allocated a unique trial identification number to preserve patient confidentiality and enable the study to be double-blinded.

1.3.3 Medication preparation and blinding/unblinding procedures:

Both liraglutide and placebo-control were packaged and labelled with a unique identification number (in keeping with the European Unions Good Manufacturing Practice for Medicinal Product guidelines) in by the manufacturer (Novo Nordisk Ltd), to the extent that the receiving trial site was blinded to the study drug

For peer review only - http://bmjopen.bmj.com/site/about/guidelines.xhtml

throughout the duration of the trial. Sealed parcels (containing electronic information) were sent with each drug package for the attention of the unblinded members of the central trial management group (TMG) (nominated statistician, PG and database programmer, PM, to ensure a) safe delivery of the correct drug and b) blinding of the treatment allocation from the remainder of the TMG and the trial patient. An independent unblinding service (24/7) was provided by the Medical toxicology and Information services, Guys hospital (London, UK), throughout the duration of the trial.

Unblinding of treatment only take place if the identity of the allocated study medication was necessary for patient safety and care. If a serious adverse event (SAE) was deemed unexpected and possibly, probably or definitely related to liraglutide (i.e. suspected unexpected serious adverse reaction = SUSAR), a clinical member of the TMG was unblinded to the medication to evaluate causality. Subsequently, the event was either labelled as an unrelated SAE (for patients receiving placebo) or a SUSAR (for patients receiving liraglutide). The latter were reported to the MHRA and the NRES, and only if patient safety was jeopardised was the study medication discontinued and the treating clinician/patient informed.

1.3.4 Adverse event (AE) reporting and analysis

The reporting period for AEs commenced at screening visit 1 and continued until follow-up visit 8. SAEs were reported until day 336 (week 48) of the trial treatment and for 30 days post-EOT. All SAEs and adverse reactions were evaluated by the

For peer review only - http://bmjopen.bmj.com/site/about/guidelines.xhtml

investigators and recorded. The National Cancer Institute's common terminology criteria for AEs (CTCAE, version 4.02, 2010) was used to grade each AE. The central trial office (CRCTU, Birmingham) kept detailed records of all AEs reported (nature, onset, duration, severity, outcome) and performed an evaluation with respect to seriousness, causality and expectedness. Interim analysis of safety data was performed and presented to the independent data management committee (DMC) on a 6-montly basis. The unblinded DMC advised accordingly with regards to the trial conduct and specifically whether extra/new data monitoring was required for the remainder of the trial. The DMC operated in accordance with a trial specific charter based upon the template created by the Damocles Group. Specific attention was given to AEs related to the thyroid (measures of blood calcitonin, TSH and physical examination) and pancreas (blood amylase, symptom recognition for pancreatitis), in light of previous non-human (rodents) and post-marketing human safety data (in patients with diabetes), respectively (45, 46).

1.3.5 Study visit overview

The LEAN trial involved 8 patient-related visits at their nearest trial site. The study was divided into four stages: (1) screening, enrolment, randomisation and baseline investigations (visits 1 and 2, over a maximum period of 14 days), (2) 336 days of study treatment (visits 3,4,5 and 6, over 48 weeks), (3) Primary end-point assessment including liver biopsy (visit 7, within 1 day of the EOT), and (4) post-treatment follow-up assessment (visit 8, 12 weeks after EOT). If the trials

For peer review only - http://bmjopen.bmj.com/site/about/guidelines.xhtml

investigating team or the trial participant suspected an adverse event, an unscheduled visit was arranged within 24 hours.

The schedule for the study visits and data collection is summarised in **Table 2**. All subjects were asked to attend each visit fasted from eating or drinking (with exception of water) for a minimum of 8 hours prior to each visit. A follow-up liver biopsy (i.e. primary end-point) was obtained under ultrasound-guidance after completion of 48 weeks study treatment. Wherever possible, a 16-gauge biopsy needle and a specimen length of a minimum of 15 mm were preferred. The liver tissue was prepared at the local trial sites in preparation for histological assessment (under light microscopy) at the central trial site at the Queen Elizabeth University Hospital Birmingham. On receipt, the two central 'blinded' central histopathologists recorded the size and quality of the histology slides. A minimum of four unstained slides was available for each liver biopsy to enable repeat staining (H&E, haematoxylin van Gieson, Ubiquitin) to ensure adequate quality for interpretation.

1.3.6 Storage of trial samples

Liver biopsy tissue specimens were collected, paraffin-fixed and stored at the diagnostic archive of the department of cellular pathology (University Hospital Birmingham). Serum and plasma samples collected at visit 1 (screening), visit 4, visit 5, visit 7 (EOT) and visit 8 (12 weeks post EOT) were stored frozen in 0.5-1.0ml aliquots at -80°C at the Institute of Biomedical Research, University of Birmingham. Where possible, additional blood (buffer coat) were obtained at visits 1 and 7 for

For peer review only - http://bmjopen.bmj.com/site/about/guidelines.xhtml

future DNA extraction and stored at -80°C. Both specimen storage banks hold a licence from the Human Tissue Authority to store tissue for research purposes.

1.3.7 Treatment compliance

Treatment compliance was monitored by a review of the used pre-filled treatment pens, participant injection sites, and the participants self-filled 'standardised treatment and clinical events booklet' at each study visit. The latter provided written evidence of dosage, time and date when each patient administers the study drug.

1.3.8 Data handling, quality assurance, record keeping and retention

Data management was undertaken according to the standard operating procedures (SOPs) of the CRCTU at the University of Birmingham, UK. The CRCTU was fully compliant with the Data Protection Act 1998 and the International Conference on Harmonisation Good Clinical Practice (ICH GCP). The CRCTU was responsible for monitoring the trial and providing annual reports to the MHRA. The trial was registered with the Data Protection Act website at the University of Birmingham. Participant identifiable data were shared only within the clinical team on a need-to-know basis to provide clinical care, and to ensure good and appropriate follow-up. Patient identifiable data were also shared with approved auditors from the NRES, Competent authorities (including MHRA, EMA and FDA), Sponsor (University of Birmingham), NHS R&D departments and the primary care practitioner. All LEAN participants provided specific written-consent at trial entry to enable data to be

For peer review only - http://bmjopen.bmj.com/site/about/guidelines.xhtml

shared with the above. Otherwise, confidentiality was maintained throughout the trial and thereafter. On completion of the trial, data will be transferred to a secure archiving facility at the University of Birmingham, where data will be held for a minimum of 10 years and then destroyed.

1.3.9 Case Report Forms

Case report forms included baseline/follow-up medical history and physical examinations to capture co-morbidities and concomitant medications in the trials electronic database. Other case report forms incorporated in the electronic database included: laboratory tests and questionnaire results were recorded for visit 1 (eligibility criteria) through to visit 8; safety monitoring during the treatment follow-up periods; central site histopathology reports of liver biopsy specimens; specialist non-invasive markers of liver disease; adverse event reporting; and study drug dispensing forms for study treatment adherence and accountability.

1.3.10 Sponsorship, Indemnity and Monitoring

The University of Birmingham acted as the sponsor of the trial. As sponsor the University was responsible for the general conduct of the study and indemnified the trial centre against any claims, arising from any negligent act or omission by the University in fulfilling the sponsor role in respect of the study. Both on-site and off-site monitoring of the trial were performed as per the LEAN Trial Quality Management Plan.

For peer review only - http://bmjopen.bmj.com/site/about/guidelines.xhtml

1.3.11 Sources of funding

The trial was funded by the Wellcome Trust (Clinical Research Fellowship awarded to MJA, 200), Novo Nordisk Ltd (free study drug supply, educational grant) and the NIHR liver BRU.

For peer review only

For peer review only - http://bmjopen.bmj.com/site/about/guidelines.xhtml

1.4 Trial status

Recruitment into the LEAN trial commenced in August 2010 and ended in May 2013, with 52 patients (104% of target enrolment) randomised from 5 trial sites (Birmingham 31; Nottingham 12; Hull 6; Leeds 3; Southampton 0). This number is 2 more than planned so as to allow all participants that had registered/consented and found to be eligible to participate in the trial. **Supplementary figure 2** summarises the recruitment rate throughout the trial. A total of 73 patients were registered for the trial, 21 (29%) of whom were not eligible or withdrew consent before randomisation to the trial. Failure to meet the histological inclusion criteria (after central histopathology review) was the most frequent reason for ineligibility. The treatment follow-up of LEAN participants is currently ongoing and the last trial visit of the last participant is due to take place in July 2014.

For peer review only

For peer review only - http://bmjopen.bmj.com/site/about/guidelines.xhtml

1.5 Discussion

Compliance with the trial protocol and safety profile of liraglutide was reviewed on a bi-annual basis by an independent DMC, and no concerns were raised.

1.5.1 Challenges in trial design

Despite recent advances in non-invasive markers of liver injury (e.g. transient elastography, serum fibrosis markers), liver biopsy remains the recommended method for assessment of disease activity for phase II/III trials (33). Liver biopsy is not without its limitations (such as sampling error, invasive nature and patient reluctance for repeat sampling (47)), but until the accuracy of serial measurements of non-invasive markers have been formally validated, it will be required for trials in NASH. The LEAN trial has attempted to minimise these limitations. First, liver biopsies (<6 months of screening) performed for routine NHS diagnostic purposes were incorporated into the eligibility criteria and utilised as the baseline comparator, rather than performing two biopsies for the sole purposes of the trial. This approach is widely accepted in trials of NASH. Second, all of the liver biopsies (baseline, primary end-point) underwent a blinded central review by two independent expert liver histopathologists (RB, SGH) at the one site, ensuring that only patients with 'definite' NASH were recruited to the trial and reducing intra/inter-assessor variability, which has previously been reported between trial sites (48).

For peer review only - http://bmjopen.bmj.com/site/about/guidelines.xhtml

In 2011, Sanyal and colleagues (update from AASLD research workshop, 2009) published expert guidance on clinical trial design in patients with NASH (33). Even though the LEAN trial design preceded this workshop, the definition of NASH and the outcome measures were in keeping with their recommendations. Patients with NASH have a higher risk of liver-related mortality than those with simple hepatic steatosis (+/- mild inflammation) (49, 50). Due to the long time-span of NASH progression (i.e. 10-20 years) to end-stage liver failure/death it is impractical to perform therapeutic trials with mortality as the primary outcome measure. Therefore, we elected to use disappearance of NASH with no worsening of fibrosis as 'surrogate' primary end-point in LEAN. With this in mind, 48-weeks treatment duration was selected, rather than 2-5 years, which would be required if we were aiming to demonstrate significant improvements in fibrosis. NAS has been incorporated as a secondary outcome measure (inc. the individual components of NAS) to represent disease activity (31), rather than as the primary outcome as previously reported (48, 51). NAS alone was not originally designed to infer absence or presence of NASH (52), which we deemed a more meaningful clinical outcome.

We elected to recruit patients with and without type 2 diabetes to enhance recruitment rates and broaden the safety data in liraglutide in NASH, but under the provision that patients with diabetes must have moderate glycaemic control (HbA1c <9.0%) on diet +/- oral hypoglycaemic medications (with the exception of TZDs and other potential confounders i.e. GLP-1 based therapy) prior to trial entry. In the knowledge that diabetes is a potential confounding factor, randomisation was

For peer review only - http://bmjopen.bmj.com/site/about/guidelines.xhtml

programmed to stratify for diabetes to ensure equal numbers in each treatment arm.

Efficient recruitment for clinical trials in NASH remains a challenge, mainly due to the requirement for liver biopsy, which has been compounded by the recent uptake of non-invasive markers (e.g. transient elastography) in the UK resulting in a decline in liver biopsy requests in some recruiting centres (37).

1.5.2 Safety profile of liraglutide

Prior to the start of the LEAN trial, the summary of product characteristics (SmPc) for liraglutide (Victoza®) stated special warnings and precautions for use in moderate/severe renal impairment, moderate/severe congestive heart failure (NHYA class III/IV), pre-existing thyroid disease and in patients at risk of pancreatitis/pancreatic carcinoma (53). In turn, the eligibility criteria (**Table 1**) reflected these warnings by excluding patients with or at risk of such. In particular, based on the pre-clinical incidence of thyroid C-cell tumours in rodent models and the manufacturers 'black box' warning in humans (53), all patients with a personal history/family history of thyroid carcinoma, multiple endocrine neoplasia syndrome type 2 and/or abnormal thyroid examination (goiter, nodules) were excluded from the trial. In addition, serum calcitonin, TFTs and clinical thyroid examination were monitored throughout the trial as a precautionary measure.

For peer review only - http://bmjopen.bmj.com/site/about/guidelines.xhtml

In keeping with both US Food and Drug Administration (FDA) (54) and European Medicines Agency (EMA) (55) recommendations, all patients in LEAN were given written/verbal advise about the risks and carefully monitored for signs and symptoms indicative of pancreatitis. In Marsh 2013, a small study (n=8) by Butler et al reported pancreatic cellular changes, consistent with pancreatic duct metaplasia, in organ donors who had received GLP-1 therapy for diabetes prior to death (56). In response in July 2013, the EMA's committee of Medicinal Products for Human Use (CHMP) critically appraised the study and all other non-clinical/clinical data available, and concluded that the current evidence did not confirm an increased risk of pancreatic adverse events with GLP-1 based therapies (57). Subsequently, the current safety measures adopted by the LEAN trial will continue until further information is made available.

1.5.3 Summary

To the best of our knowledge, the LEAN trial is the first multi-centre, double-blinded, placebo-controlled RCT designed to investigate whether the long-acting GLP-1 analogue, liraglutide, is safe and improves liver histology in overweight patients with NASH. The enrolment of the required sample size was completed in May 2013 and the final results are expected by the end of 2014. The full LEAN protocol (version 7.0) can be obtained from the NIHR liver biomedical research unit and CRCTU at the University of Birmingham (LEAN@trials.bham.ac.uk).

For peer review only

For peer review only - http://bmjopen.bmj.com/site/about/guidelines.xhtml

Generic Exclusion criteria:

1. Refusal or lacks capacity to give informed consent to participate in the trial
2. Participation in any clinical trial of an investigational therapy or agent within 3 months of randomisation
3. Patient (or carer) deemed not competent at using the correct site and technique for subcutaneous injection of the trial treatment (containing dummy drug on practice)
4. NAFLD Activity Score (NAS) < 3 on liver biopsy
5. Child's B or C cirrhosis
6. Past medical history of multiple drug allergies (defined as anaphylactoid drug reactions in >2 drug groups)
7. Presence of any acute/chronic infections or illness that at the discretion of the chief investigator might compromise the patient's health and safety in the trial
8. Pregnancy or breastfeeding
9. Women, of child-bearing age, who are not willing to practise effective contraception (i.e. barrier, oral contraceptive pill, impenon or past medical history of hysterectomy) for the 48 week duration of the trial and for one-month after the last administration of the drug.
10. Men, sexually active with women of child-bearing age, who are not willing to practise effective contraception for the 48 week duration of the trial and for one-month after the last administration of the drug.
11. Liver disease of other aetiologies (i.e. drug-induced, viral hepatitis, autoimmune hepatitis, PBC, PSC, haemochromatosis, A1AT deficiency, Wilsons disease)
12. Past medical/surgery history of; Gastric bypass surgery, orthotopic liver transplant (OLT) or listed for OLT, hepatocellular, pancreatic, thyroid carcinoma, multiple endocrine neoplasia syndrome type 2 (MEN 2), acute or chronic pancreatitis, and total parenteral nutrition within 6 months of randomisation.
13. Diagnosis of malignancy within the last 3 years (with the exception of treated skin malignancies)
14. Hepatocellular Carcinoma: dysplastic or intermediate nodules to be excluded. Borderline cases to be discussed at Birmingham's tertiary hepato-biliary multidisciplinary team (MDT) meeting. Regenerative and other nodules to be included at the discretion of the chief investigator and the MDT.
15. Family history of medullary thyroid carcinoma
16. Clinical evidence of decompensated chronic liver disease: radiological or clinical evidence of ascites, current or previous hepatic encephalopathy and evidence of portal hypertensive haemorrhage or varices on endoscopy
17. Abnormal clinical examination of thyroid (i.e. unexplained goitre or palpable nodules)
18. ALT or AST > 10 x upper limit of normal
19. Average alcohol consumption/week male >21 (approx. 210g), female >14 units (approx. 140g) within the last 5 years.
20. >5% weight loss since the diagnostic liver biopsy was obtained.
21. Recent (within 3 months of the diagnostic liver biopsy or screening visit) or significant change (as judged by the chief investigator) in dose of the following drugs: Inducers of hepatic steatosis (steroids (oral/intravenous), methotrexate, amiodarone), orlistat and/or multi-vitamins/vitamin E (containing >200% recommended daily amount; >30mg/day)
22. Known positivity for antibody to Human Immunodeficiency virus (HIV)
23. Serum creatinine >150 μmol/L or currently being treated with renal replacement therapy (i.e. Haemodialysis or Peritoneal Dialysis)

Specific exclusion criteria for subjects with T2D:

1. Current or previous insulin therapy, with exception of previous short-term insulin treatment in connection with intercurrent illness is allowed (≥ 3 months prior to screening), at the discretion of the chief investigator.
2. Subjects receiving thiazolidinediones (TZDs), dipeptidy peptidase (DPP) IV inhibitors and other GLP-1 based therapies (i.e. exenatide)
3. HbA1c ≥ 9.0%
4. Recurrent major hypoglycaemia or hypoglycaemic unawareness as judged by the chief investigator

Table 1: Exclusion criteria for LEAN trial

For peer review only - http://bmjopen.bmj.com/site/about/guidelines.xhtml

	Screening		Treatment (TD, treatment day)					Follow-up
	Visit 1 (Max -14 days to TD1)	Visit 2 (1 day prior to TD1)	Visit 3 (TD 28)	Visit 4 (TD 84)	Visit 5 (TD 168)	Visit 6 (TD 252)	Visit 7 (1 Day + TD 336/ End of Treatment [EOT])	Visit 8 (12 weeks after EOT)
Informed consent	X							
Clinical assessment [1]	X		X	X	X	X	X	X
Vital Signs [2]	X		X	X	X	X	X	X
ECG/Urine Dipstix	X			X	X	X	X	X
Standard blood tests [3]	X		X	X	X	X	X	X
Screening blood tests [4]	X							
Lipid profile<br>Serum insulin	X			X	X		X	X
OGTT (non-diabetics only)	X						X	
Non-invasive fibrosis markers [5]	X						X	X
Metabolic sub-studies [6]		X		X				
Questionnaires [7]	X						X	X
**Liver biopsy**	- [8]						X	

For peer review only

For peer review only - http://bmjopen.bmj.com/site/about/guidelines.xhtml

Adverse/ Clinical events [9]			**X**	**X**	**X**	**X**	**X**	**X**
Study medication dispensed		**X** [10]	**X**	**X**	**X**	**X**		

Table 2. Trial schedule of data collection

Figure legends:

Figure 1: Schematic of LEAN trial design.
Eligible participants are randomly assigned to 48 weeks treatment of once-daily (OD) subcutaneous injections (SC) of either 1.8mg liraglutide or placebo-control. Both the trial investigators and the participants are blinded to drug allocation.

Figure 2: Histological inclusion criteria for LEAN trial. Liver biopsy sections (actual magnification 400X). **[A – B] 'Uncertain' NASH - not eligible for LEAN**: **[A]** H&E stain highlights fat, inflammation and some pale cells, however **[B]** ubiquitin immunohistochemistry does not identify any Mallory Denk bodies (no confirmed ballooning). **[C – D] 'Uncertain' NASH - eligible for LEAN**: **[C]** H&E stain highlights fat, inflammation and pale cells, but with no obvious Mallory Denk bodies. However, ubiquitin staining **[D]** is positive (confirming ballooned hepatocytes). **[E – F] 'Definite' NASH - eligible for LEAN**: Both H&E and ubiquitin staining highlight fat, lobular inflammation and widespread ballooned hepatocytes. Black arrows highlight Mallory Denk bodies.

Table 1: LEAN trial Exclusion criteria
Patients who met any of the criteria (*listed* above) at the screening visit were excluded from trial participation

Table 2: Data collection schedule
[1] Clinical assessment: complete history/examination (visit 1), focussed history/examination (visits 2-8). **[2]** Vital signs: HR, BP, weight, Height, waist:hip circumference, body temperature, SaO_2, RR. **[3]** Standard fasting blood tests: FBC, U+E, LFTs, INR, TFTs, glucose and HbA1c (*except visit 3*). **[4]** Screening blood tests: HBsAg, HCV Ab , AMA/ASA/immunoglobulins, Ferritin/Transferrin saturation, Caeruloplasmin, α1AT, AFP. **[5]** FibroMAX panel (FibroTest, SteatoTest, NashTest), ELF tests and transient elastography (Fibroscan; optional depending on availability). **[6]** Optional metabolic sub-study: 2-step hyperinsulinaemic euglycaemic clamp with stable isotope studies and adipose microdialysis. **[7]** Questionnaires: AUDIT, Block Brief 2000 FFQ, HR-QOL (SF-36v2). **[8]** Diagnostic liver biopsy performed as part of standard NHS care ≤6 months of screening visit 1. Two independent liver histopathologists will review the liver biopsy to assess whether the patients meets the histological inclusion criteria. Adverse Events/bloods and Clinical Events will be monitored continuously until completion of follow up and 30 days after. Calcitonin

For peer review only - http://bmjopen.bmj.com/site/about/guidelines.xhtml

and AFP levels will be measured at visits 1, 5, 7 and 8. **[10]** If the study patient meets the eligibility criteria, he/she will be randomised at visit 2 to receive liraglutide (Victoza®) or placebo. The allocated blinded study treatment will be dispensed at visit.

Supplementary Table 1: Trial proforma for the histopathological assessment of pre- and post-treatment liver biopsies.
Two independent liver histopathologists will perform the histological assessments on the pre and post treatment liver biopsies. *In the event that one histopathologist reports the diagnosis of NASH as 'uncertain,' then a joint review will take place to determine if the participant is eligibly for randomization. If both histopathologists regard the case as "uncertain", this is classed as "no" for eligibility purposes.

Supplementary Figure 1: Recruitment rate for LEAN trial
In total 52 patients were recruited over a period of 32 months

<u>Author contributions:</u>

MJA, SG, JWT, and PNN (Chief Investigator) had the original concept of the LEAN trial. MJA, SG, JWT, and PNN designed the LEAN trial and wrote/reviewed all protocol versions. RB and SGH designed the proforma used for recording histopathological findings and carried out the central histopathology review of all pre- and post-treatment liver biopsies**.** MJA and DB (senior trials coordinator) submitted all REC, MHRA and local R&D applications. MJA, PNN, PG and DS devised the statistical plan. PG prepared the bi-annual DMC reports. MJA, DB, DH, and KG wrote/designed the patient information sheets, external trial information and patient CRFs. MJA wrote the manuscript and all authors reviewed the final version. MJA and PNN are guarantors.

Other members of the **LEAN trial group** that have been instrumental in the conduct of the trial to date:

For peer review only - http://bmjopen.bmj.com/site/about/guidelines.xhtml

Queen Elizabeth University Hospital Birmingham/NIHR Liver BRU/CRUKCTU (Birmingham, UK): Manpreet Wilku, Christine Russell, Salma Iqbal, Dr Christopher Corbett, Michelle Yun Kyong Lee and nursing staff at the WTCRF.

Nottingham University Hospitals NHS Trust/ Nottingham Digestive Diseases BRU (Nottingham, UK): Professor Guru P. Aithal (Principal Investigator), Maggie Nicholls, Susanne Henry.

Hull Royal Infirmary (Hull, UK): Dr George Abouda (Principal Investigator), Martin Lewis, Erica Dixon.

St James Hospital (Leeds, UK): Dr Mark Aldersley (Principal Investigator), Samantha Sharman, Rebecca Bishop, Dr Waleed Fateen.

Southampton General Infirmary (Southampton, UK): Dr Kate Nash (Principal Investigator), Julie Mitchell, Amy King, Lisa Fraser.

Acknowledgements:

The LEAN trial would like to thank the Data Management Committee (DMC) consisting of Professor Peter Hayes (DMC Chair; independent Liver expert), Sarah Brown (Independent Senior Statistician) and Dr Jude Oben (Independent Liver expert) for their time and input. The LEAN trial is funded by Wellcome Trust (Clinical Research Fellowship awarded to MJA), Novo Nordisk Ltd (educational grant, free supply of trial drugs) and the NIHR Liver BRU.

Conflict of Interests:

PNN and MJA have received an educational grant and free trial drug supply from Novo Nordisk for conduct of the LEAN trial of liraglutide in NASH. PNN has received

For peer review only - http://bmjopen.bmj.com/site/about/guidelines.xhtml

honoraria for lectures given on behalf of Novo Nordisk. SCLG has served on advisory boards for Novo Nordisk, Eli Lilly, Sanofi Aventis and Takeda, and has received honoraria for lectures given on behalf of Novo Nordisk, Eli Lilly, Sanofi Aventis, Takeda and GSK. JWT, DB, DH, KG, DS and PG have no conflict of interests to declare.

Data sharing

The full (detailed) clinical trials protocol is available on request at LEAN@trials.bham.ac.uk.

For peer review only

For peer review only - http://bmjopen.bmj.com/site/about/guidelines.xhtml

References:

1. Armstrong MJ, Houlihan DD, Bentham L, et al. Presence and severity of non-alcoholic fatty liver disease in a large prospective primary care cohort. J Hepatol 2012;56:234-240.
2. Bellentani S, Tiribelli C, Saccoccio G, et al. Prevalence of chronic liver disease in the general population of northern Italy: the Dionysos Study. Hepatology 1994;20:1442-1449.
3. Browning JD, Szczepaniak LS, Dobbins R, et al. Prevalence of hepatic steatosis in an urban population in the United States: impact of ethnicity. Hepatology 2004;40:1387-1395.
4. Bellentani S, Bedogni G, Miglioli L, et al. The epidemiology of fatty liver. Eur J Gastroenterol Hepatol 2004;16:1087-1093.
5. Williams CD, Stengel J, Asike MI, et al. Prevalence of nonalcoholic fatty liver disease and nonalcoholic steatohepatitis among a largely middle-aged population utilizing ultrasound and liver biopsy: a prospective study. Gastroenterology 2011;140:124-131.
6. Bugianesi E, Leone N, Vanni E, et al. Expanding the natural history of nonalcoholic steatohepatitis: from cryptogenic cirrhosis to hepatocellular carcinoma. Gastroenterology 2002;123:134-140.
7. Charlton MR, Burns JM, Pedersen RA, et al. Frequency and outcomes of liver transplantation for nonalcoholic steatohepatitis in the United States. Gastroenterology 2011;141:1249-1253.
8. Baggio LL, Drucker DJ. Biology of incretins: GLP-1 and GIP. Gastroenterology 2007;132:2131-2157.
9. Deacon CF, Johnsen AH, Holst JJ. Degradation of glucagon-like peptide-1 by human plasma in vitro yields an N-terminally truncated peptide that is a major endogenous metabolite in vivo. J Clin Endocrinol Metab 1995;80:952-957.
10. Knudsen LB, Nielsen PF, Huusfeldt PO, et al. Potent derivatives of glucagon-like peptide-1 with pharmacokinetic properties suitable for once daily administration. J Med Chem 2000;43:1664-1669.
11. Astrup A, Rössner S, Van Gaal L, et al. Effects of liraglutide in the treatment of obesity: a randomised, double-blind, placebo-controlled study. Lancet 2009;374:1606-1616.
12. Jendle J, Nauck MA, Matthews DR, et al. Weight loss with liraglutide, a once-daily human glucagon-like peptide-1 analogue for type 2 diabetes treatment as monotherapy or added to metformin, is primarily as a result of a reduction in fat tissue. Diabetes Obes Metab 2009;11:1163-1172.
13. Buse JB, Rosenstock J, Sesti G, et al. Liraglutide once a day versus exenatide twice a day for type 2 diabetes: a 26-week randomised, parallel-group, multinational, open-label trial (LEAD-6). Lancet 2009;374:39-47.
14. Garber A, Henry R, Ratner R, et al. Liraglutide versus glimepiride monotherapy for type 2 diabetes (LEAD-3 Mono): a randomised, 52-week, phase III, double-blind, parallel-treatment trial. Lancet 2009;373:473-481.
15. Marre M, Shaw J, Brändle M, et al. Liraglutide, a once-daily human GLP-1 analogue, added to a sulphonylurea over 26 weeks produces greater

For peer review only - http://bmjopen.bmj.com/site/about/guidelines.xhtml

improvements in glycaemic and weight control compared with adding rosiglitazone or placebo in subjects with Type 2 diabetes (LEAD-1 SU). Diabet Med 2009;26:268-278.
16. Nauck M, Frid A, Hermansen K, et al. Efficacy and safety comparison of liraglutide, glimepiride, and placebo, all in combination with metformin, in type 2 diabetes: the LEAD (liraglutide effect and action in diabetes)-2 study. Diabetes Care 2009;32:84-90.
17. Russell-Jones D, Vaag A, Schmitz O, et al. Liraglutide vs insulin glargine and placebo in combination with metformin and sulfonylurea therapy in type 2 diabetes mellitus (LEAD-5 met+SU): a randomised controlled trial. Diabetologia 2009;52:2046-2055.
18. Zinman B, Gerich J, Buse JB, et al. Efficacy and safety of the human glucagon-like peptide-1 analog liraglutide in combination with metformin and thiazolidinedione in patients with type 2 diabetes (LEAD-4 Met+TZD). Diabetes Care 2009;32:1224-1230.
19. Mayor S. NICE approves liraglutide for diabetic patients not achieving glucose control. BMJ 2010;341:c5062.
20. Ben-Shlomo S, Zvibel I, Shnell M, et al. Glucagon-like peptide-1 reduces hepatic lipogenesis via activation of AMP-activated protein kinase. Journal of Hepatology 2011;54:1214-1223.
21. Ding X, Saxena NK, Lin S, et al. Exendin-4, a glucagon-like protein-1 (GLP-1) receptor agonist, reverses hepatic steatosis in ob/ob mice. Hepatology 2006;43:173-181.
22. Mells JE, Fu PP, Sharma S, et al. Glp-1 analog, liraglutide, ameliorates hepatic steatosis and cardiac hypertrophy in C57BL/6J mice fed a Western diet. Am J Physiol Gastrointest Liver Physiol 2012;302:G225-235.
23. Gupta NA, Mells J, Dunham RM, et al. Glucagon-like peptide-1 receptor is present on human hepatocytes and has a direct role in decreasing hepatic steatosis in vitro by modulating elements of the insulin signaling pathway. Hepatology 2010;51:1584-1592.
24. Svegliati-Baroni G, Saccomanno S, Rychlicki C, et al. Glucagon-like peptide-1 receptor activation stimulates hepatic lipid oxidation and restores hepatic signalling alteration induced by a high-fat diet in nonalcoholic steatohepatitis. Liver Int 2011;31:1285-1297.
25. Sharma S, Mells JE, Fu PP, et al. GLP-1 Analogs Reduce Hepatocyte Steatosis and Improve Survival by Enhancing the Unfolded Protein Response and Promoting Macroautophagy. PLoS ONE 2011;6:e25269.
26. Ellrichmann M, Vollmer K, Schrader H, et al. Sustained virological response during exenatide treatment in a patient with hepatitis C and nonalcoholic steatohepatitis. Am.J.Gastroenterol. 2009;104:3112-3114.
27. Tushuizen ME, Bunck MC, Pouwels PJ, et al. Incretin mimetics as a novel therapeutic option for hepatic steatosis. Liver Int 2006;26:1015-1017.
28. Kenny PR, Brady DE, Torres DM, et al. Exenatide in the treatment of diabetic patients with non-alcoholic steatohepatitis: a case series. The American Journal of Gastroenterology 2010;105:2707-2709.
29. Buse JB, Klonoff DC, Nielsen LL, et al. Metabolic effects of two years of exenatide treatment on diabetes, obesity, and hepatic biomarkers in patients with type 2 diabetes: an interim analysis of data from the open-label, uncontrolled extension of three double-blind, placebo-controlled trials. Clin Ther 2007;29:139-153.

For peer review only - http://bmjopen.bmj.com/site/about/guidelines.xhtml

30. Armstrong MJ, Houlihan DD, Rowe IA, et al. Safety and efficacy of liraglutide in patients with type 2 diabetes and elevated liver enzymes: individual patient data meta-analysis of the LEAD program. Aliment Pharmacol Ther 2013;37:234-42.
31. Kleiner DE, Brunt EM, Van Natta M, et al. Design and validation of a histological scoring system for nonalcoholic fatty liver disease. Hepatology 2005;41:1313-1321.
32. Health Canada. Standards for clinical trials in type 2 diabetes in Canada; 2007. Available at: http://www.hc-sc.gc.ca.
33. Sanyal AJ, Brunt EM, Kleiner DE, et al. Endpoints and clinical trial design for nonalcoholic steatohepatitis. Hepatology 2011;54:344-353.
34. Ishak K, Baptista A, Bianchi L, et al. Histological grading and staging of chronic hepatitis. J.Hepatol. 1995;22:696-699.
35. Brunt EM, Kleiner DE, Wilson LA, et al. Portal chronic inflammation in nonalcoholic fatty liver disease (NAFLD): a histologic marker of advanced NAFLD-Clinicopathologic correlations from the nonalcoholic steatohepatitis clinical research network. Hepatology 2009;49:809-820.
36. Rosenberg WMC, Voelker M, Thiel R, et al. Serum markers detect the presence of liver fibrosis: a cohort study. Gastroenterology 2004;127:1704-1713.
37. Armstrong MJ, Corbett C, Hodson J, et al. Operator training requirements and diagnostic accuracy of Fibroscan in routine clinical practice. Postgrad Med J 2013. [Epub ahead of print].
38. Ware JE. Improvements in short-form measures of health status: introduction to a series. J Clin Epidemiol 2008;61:1-5.
39. Block G, Woods M, Potosky A, et al. Validation of a self-administered diet history questionnaire using multiple diet records. J.Clin.Epidemiol. 1990;43:1327-1335.
40. Reinert DF, Allen JP. The Alcohol Use Disorders Identification Test (AUDIT): a review of recent research. Alcohol Clin.Exp.Res. 2002;26:272-279.
41. Lindor KD, Kowdley KV, Heathcote EJ, et al. Ursodeoxycholic acid for treatment of nonalcoholic steatohepatitis: results of a randomized trial. Hepatology 2004;39:770-778.
42. Ratziu V, Giral P, Jacqueminet S, et al. Rosiglitazone for nonalcoholic steatohepatitis: one-year results of the randomized placebo-controlled Fatty Liver Improvement with Rosiglitazone Therapy (FLIRT) Trial. Gastroenterology 2008;135:100-110.
43. A'Hern RP. Sample size tables for exact single-stage phase II designs. Stat Med 2001;20:859-866.
44. Aithal GP, Thomas JA, Kaye PV, et al. Randomized, placebo-controlled trial of pioglitazone in nondiabetic subjects with nonalcoholic steatohepatitis. Gastroenterology 2008;135:1176-1184.
45. Alves C, Batel-Marques F, Macedo AF. A meta-analysis of serious adverse events reported with exenatide and liraglutide: acute pancreatitis and cancer. Diabetes Res Clin Pract 2012;98:271-284.
46. Franks AS, Lee PH, George CM. Pancreatitis: a potential complication of liraglutide? Ann Pharmacother 2012;46:1547-1553.
47. Bravo AA, Sheth SG, Chopra S. Liver biopsy. N Engl J Med 2001;344:495-500.
48. Sanyal AJ, Chalasani N, Kowdley KV, et al. Pioglitazone, vitamin E, or placebo for nonalcoholic steatohepatitis. N Engl J Med 2010;362:1675-1685.

For peer review only - http://bmjopen.bmj.com/site/about/guidelines.xhtml

49. Ekstedt M, Franzén LE, Mathiesen UL, et al. Long-term follow-up of patients with NAFLD and elevated liver enzymes. Hepatology 2006;44:865-873.
50. Söderberg C, Stål P, Askling J, et al. Decreased survival of subjects with elevated liver function tests during a 28-year follow-up. Hepatology 2010;51:595-602.
51. Promrat K, Kleiner DE, Niemeier HM, et al. Randomized controlled trial testing the effects of weight loss on nonalcoholic steatohepatitis. Hepatology 2010;51:121-129.
52. Brunt EM, Kleiner DE, Wilson LA, et al. Nonalcoholic fatty liver disease (NAFLD) activity score and the histopathologic diagnosis in NAFLD: distinct clinicopathologic meanings. Hepatology 2011;53:810-820.
53. European Medicines Agency E. Summary of Product Characteristics (SmPc) for Victoza. Available at: http://www.ema.europa.eu/ema/index.jsp?curl=pages/medicines/human/medicines/001026/human_med_001137; 2012.
54. US Food and Drug Administration F. Victoza approval package. FDA/Centre for Drug Evaluation and Research. Available at: http://www.accessdata.fda.gov/drugsatfda_docs/nda/2010/022341s000TOC.cfm. 2013.
55. European Medicines Agency E. European public assessment report (EPAR) for Victoza. EMA/Committee for Medicinal Products for Human Use, Available at: http://www.ema.europa.eu/ema/index.jsp?curl=pages/medicines/human/medicines/001026/human_med_001137; 2009.
56. Butler AE, Campbell-Thompson M, Gurlo T, et al. Marked expansion of exocrine and endocrine pancreas with incretin therapy in humans with increased exocrine pancreas dysplasia and the potential for glucagon-producing neuroendocrine tumors. Diabetes 2013;62:2595-2604.
57. CHMP EMA. Investigation into GLP-1 based diabetes therapies concluded [Press Release July 2013]. Available at: http://www.ema.europa.eu/ema/index.jsp?curl=pages/news_and_events/news/2013/07/news_detail_001856.jsp&mid=WC0b01ac058004d5c1. 2013.

For peer review only

For peer review only - http://bmjopen.bmj.com/site/about/guidelines.xhtml

Figure 1

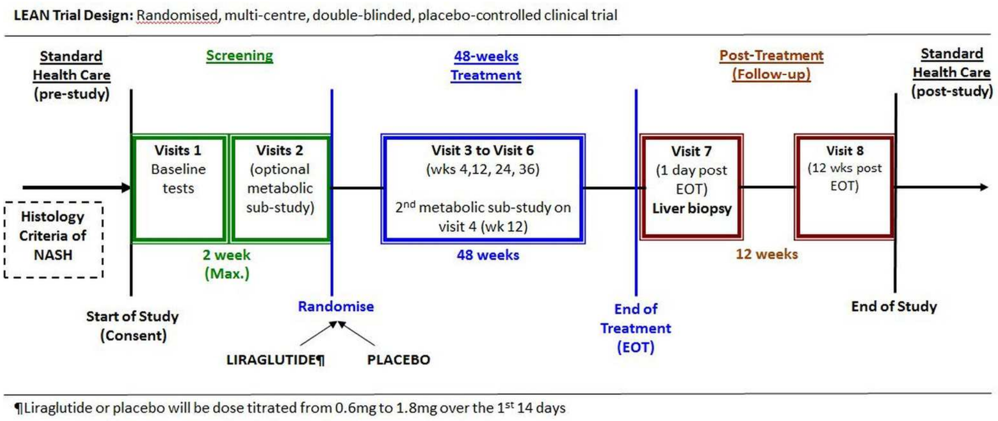

Figure 1
291x164mm (300 x 300 DPI)

review only

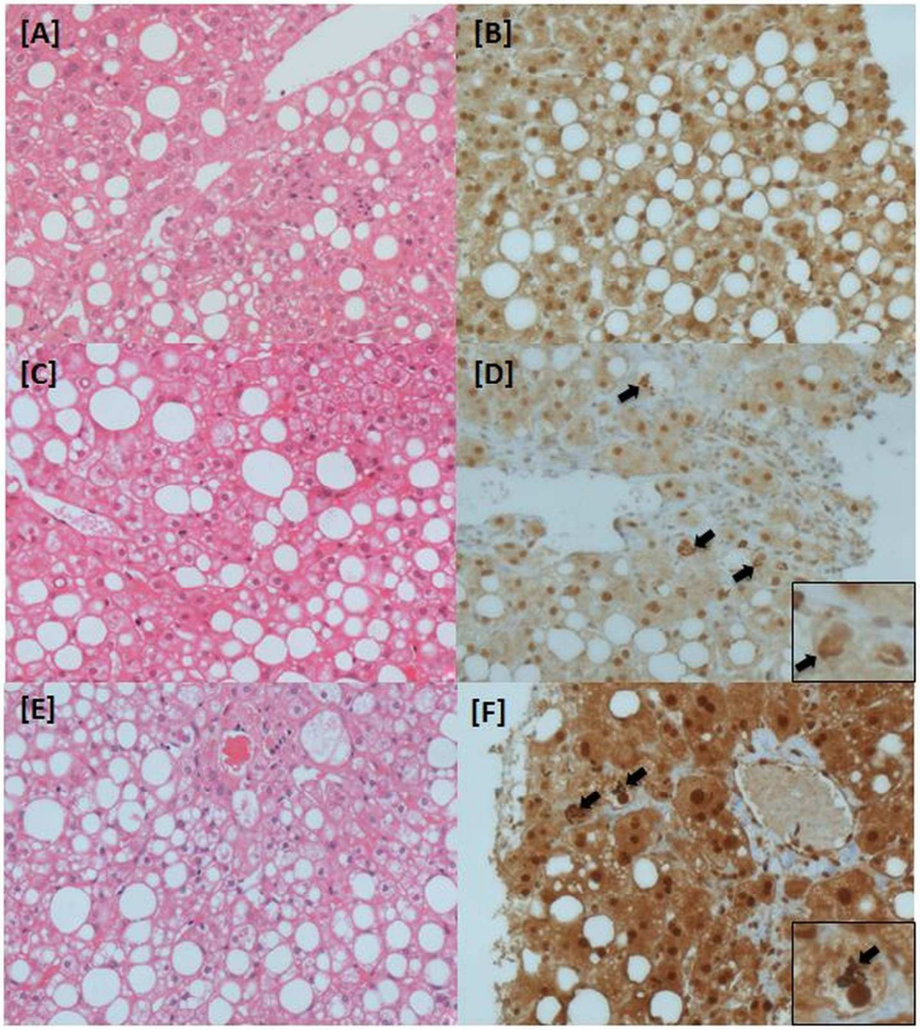

Figure 2 [A-F]

Figure 2
171x192mm (300 x 300 DPI)

**Trial participant**	Unique trial ID, date of biopsy, date of review
**Diagnosis of NASH on liver biopsy**	[ ] definite; [ ] uncertain*; no [ ]
**Quality of analysed liver biopsy**	Number of complete portal tracts __<br>Length of liver specimen (mm) __
**NAFLD Activity Score (NAS),** (Kleiner et al [31])	**Composite score (_/8)**
Steatosis, (_/3)	0=<5%;<br>1=5-33%;<br>2=>33-66%;<br>3=>66%
Lobular inflammation, (_/3)	0=No foci;<br>1=<2 foci/200x;<br>2=2-4 foci/200x;<br>3=>4 foci/ 200x
Hepatocyte Ballooning, (_/2)	0=None;<br>1=few ballooned cells;<br>2=many cells/prominent ballooning
**Portal tract changes**	
Portal inflammation (_/4) (Ishak et al [34])	0=None;<br>1=Mild, some or all portal areas;<br>2=Moderate, some or all portal areas;<br>3=Moderate/marked, all portal areas;<br>4=Marked, all portal areas.
Interface hepatitis (_/4) (Ishak et al,[34])	0=Absent;<br>1=Mild (focal, few portal areas);<br>2=Mild/moderate (focal, most portal areas)<br>3=Moderate (continuous around <50% of tracts or septa);<br>4=Severe (continuous around >50% of tracts or septa)
Ductular reaction (_/3)	1= focal in <50% of portal tracts<br>2= focal in >50% of portal tracts or prominent in <50% of portal tracts.<br>3=prominent in >50% of portal tracts.
**Kleiner Fibrosis Score (F0-F4)** (Kleiner at al, [31])	(*select one from the list*)
F0	No fibrosis
F1 [1A-1C]	Perisinusoidal OR Periportal [1A=mild, zone 3, perisinusoidal; 1B=moderate, zone 3, perisinusoidal; 1C=portal/periportal]
F2	Perisinusoidal and Portal/periportal
F3	Bridging fibrosis
F4	Cirrhosis
**Modified version of Ishak score for fibrosis [34]**	(*Select one from the list*)
0	No fibrosis
1	Zonal fibrosis involving a minority of zone 3 areas and/or portal tracts [specify whether pericellular and/or periportal]
2	Zonal fibrosis involving a majority of zone 3 areas and/or portal tracts [specify whether pericellular and/or periportal]
3	Bridging fibrosis-occasional foci [specify where central-central or central-portal or portal-portal]
4	Bridging fibrosis-widespread [specify where central-central or central-portal or portal-portal]
5	Bridging fibrosis-widespread, with occasional nodule (incomplete cirrhosis)
6	Cirrhosis – probable

Supplementary table 1

For peer review only

CONSORT 2010 checklist of information to include when reporting a randomised trial*

Section/Topic	Item No	Checklist item	Reported on page No
**Title and abstract**			
	1a	Identification as a randomised trial in the title	1
	1b	Structured summary of trial design, methods, results, and conclusions (for specific guidance see CONSORT for abstracts)	3
**Introduction**			
Background and objectives	2a	Scientific background and explanation of rationale	5-6
	2b	Specific objectives or hypotheses	5-6
**Methods**			
Trial design	3a	Description of trial design (such as parallel, factorial) including allocation ratio	8
	3b	Important changes to methods after trial commencement (such as eligibility criteria), with reasons	8-21
Participants	4a	Eligibility criteria for participants	23-25
	4b	Settings and locations where the data were collected	8-25
Interventions	5	The interventions for each group with sufficient details to allow replication, including how and when they were actually administered	9-10
Outcomes	6a	Completely defined pre-specified primary and secondary outcome measures, including how and when they were assessed	12-13
	6b	Any changes to trial outcomes after the trial commenced, with reasons	NA
Sample size	7a	How sample size was determined	18-19
	7b	When applicable, explanation of any interim analyses and stopping guidelines	25-27, 29-30
Randomisation:			
Sequence generation	8a	Method used to generate the random allocation sequence	25
	8b	Type of randomisation; details of any restriction (such as blocking and block size)	25
Allocation concealment mechanism	9	Mechanism used to implement the random allocation sequence (such as sequentially numbered containers), describing any steps taken to conceal the sequence until interventions were assigned	25
Implementation	10	Who generated the random allocation sequence, who enrolled participants, and who assigned participants to interventions	20-28
Blinding	11a	If done, who was blinded after assignment to interventions (for example, participants, care providers, those	13 onwards

For peer review only - http://bmjopen.bmj.com/site/about/guidelines.xhtml

		assessing outcomes) and how	
	11b	If relevant, description of the similarity of interventions	9-10
Statistical methods	12a	Statistical methods used to compare groups for primary and secondary outcomes	19-21
	12b	Methods for additional analyses, such as subgroup analyses and adjusted analyses	19-21
**Results**			
Participant flow (a diagram is strongly recommended)	13a	For each group, the numbers of participants who were randomly assigned, received intended treatment, and were analysed for the primary outcome	NA, protocol only
	13b	For each group, losses and exclusions after randomisation, together with reasons	‘’’
Recruitment	14a	Dates defining the periods of recruitment and follow-up	‘’’’
	14b	Why the trial ended or was stopped	‘’’’
Baseline data	15	A table showing baseline demographic and clinical characteristics for each group	‘’’’
Numbers analysed	16	For each group, number of participants (denominator) included in each analysis and whether the analysis was by original assigned groups	‘’’’
Outcomes and estimation	17a	For each primary and secondary outcome, results for each group, and the estimated effect size and its precision (such as 95% confidence interval)	‘’’’
	17b	For binary outcomes, presentation of both absolute and relative effect sizes is recommended	‘’’’
Ancillary analyses	18	Results of any other analyses performed, including subgroup analyses and adjusted analyses, distinguishing pre-specified from exploratory	‘’’’
Harms	19	All important harms or unintended effects in each group (for specific guidance see CONSORT for harms)	“”
**Discussion**			
Limitations	20	Trial limitations, addressing sources of potential bias, imprecision, and, if relevant, multiplicity of analyses	“”
Generalisability	21	Generalisability (external validity, applicability) of the trial findings	“”
Interpretation	22	Interpretation consistent with results, balancing benefits and harms, and considering other relevant evidence	“”
**Other information**			
Registration	23	Registration number and name of trial registry	3
Protocol	24	Where the full trial protocol can be accessed, if available	36
Funding	25	Sources of funding and other support (such as supply of drugs), role of funders	31

*We strongly recommend reading this statement in conjunction with the CONSORT 2010 Explanation and Elaboration for important clarifications on all the items. If relevant, we also recommend reading CONSORT extensions for cluster randomised trials, non-inferiority and equivalence trials, non-pharmacological treatments, herbal interventions, and pragmatic trials. Additional extensions are forthcoming: for those and for up to date references relevant to this checklist, see www.consort-statement.org.

For peer review only

For peer review only - http://bmjopen.bmj.com/site/about/guidelines.xhtml
